# Outcomes reported in randomized controlled trials of traditional Chinese medicine for acute pharyngitis: a scoping review

**DOI:** 10.3389/fphar.2026.1872689

**Published:** 2026-08-26

**Authors:** Danni Li, Chenyao Zhang, Yanjun Jin, Xinyao Jin, Fengwen Yang, Wentai Pang, Junhua Zhang

**Affiliations:** 1 Tianjin University of Traditional Chinese Medicine Evidence Based Medicine Center, Tianjin, China; 2 National Key Laboratory of Modern Chinese Medicine Creation, Tianjin, China

**Keywords:** acute pharyngitis, core outcome set, outcomes, scoping review, traditional Chinese medicine

## Abstract

**Purpose:**

Considerable variability exists in the outcomes reported in trials of traditional Chinese medicine (TCM) for acute pharyngitis, limiting the comparability and applicability of findings. This review aimed to systematically examine outcome selection, measurement, and reporting in these studies to inform the development of a core outcome set (COS).

**Methods:**

A systematic search was conducted to identify randomized controlled trials evaluating TCM interventions for acute pharyngitis. Data on study characteristics, outcome domains, specific outcomes, measurement instruments, and assessment time points were extracted. Outcomes were categorized using an established outcome taxonomy, and a narrative synthesis was performed to evaluate patterns of outcome reporting.

**Results:**

A total of 182 studies were included. Six broad outcome domains comprising 817 individual outcomes were identified. Substantial heterogeneity was observed in outcome selection, definitions, and measurement approaches, with most outcomes reported in only a small proportion of studies. Symptom- and sign-based outcomes were universally reported, whereas domains such as quality of life, economic evaluation, and long-term prognosis were rarely addressed. Standardized patient-reported measures and validated instruments were infrequently used. In addition, outcome reporting was often limited to short-term clinical effects, with minimal integration of safety, patient-centered, or health system–level outcomes.

**Conclusion:**

Substantial heterogeneity in outcome reporting across TCM trials for acute pharyngitis hinders cross-study comparison and evidence synthesis. This review provides an evidence base for developing a Core Outcome Set (COS) to standardize future outcome assessments and improve trial transparency.

## Introduction

Acute pharyngitis is one of the most frequently encountered disorders of the upper respiratory tract and represents a common cause of outpatient visits among both pediatric and adult populations (Pellegrino et al., 2023a). In etiological terms, viral infection is responsible for the majority of cases, and the illness is generally self-limited, while bacterial infection contributes to a smaller proportion (Pellegrino et al., 2023a). Patients with acute pharyngitis usually present with throat pain and discomfort on swallowing, often accompanied by fever, erythema of the pharynx, enlargement or exudation of the tonsils, and cervical lymph node swelling (Vincent et al., 2004). Despite its usually short clinical course, which commonly resolves within approximately 1 week, acute pharyngitis can still adversely affect patients’ short-term wellbeing and has been associated with substantial antibiotic prescribing in outpatient settings (Malmberg et al., 2026; Catic et al., 2018). According to a recent review, acute pharyngitis contributes to approximately 2%–5% of ambulatory visits in children and remains a major indication for antibiotic use in pediatric practice (Malmberg et al., 2026; Pellegrino et al., 2023b). Although the overall prognosis is favorable, a small subset of patients may subsequently develop suppurative complications, including peritonsillar abscess, otitis media, and sinusitis, underscoring the need for prompt assessment and appropriate management (Hedin et al., 2023).

Given the substantial clinical burden of acute pharyngitis and the ongoing need to reduce unnecessary antibiotic exposure, traditional Chinese medicine (TCM)-based therapeutic strategies have attracted increasing clinical and research attention in China (Yan-ning et al., 2025; Lin et al., 2024a). Modern TCM management of acute pharyngitis is usually guided by syndrome differentiation; in both clinical practice and research, Chinese patent medicines are commonly employed, while compound herbal prescriptions also remain widely used (Yan-ning et al., 2025; Lin et al., 2024a; Rong et al., 2024). Accordingly, outcome assessment in this field has become increasingly multidimensional and can be organized into domains such as Symptoms/Signs, Physical and laboratory examination, Economic evaluation, Quality of life, TCM Syndrome, and Safety outcomes (Jianxin et al., 2021), rather than relying solely on a single composite overall response measure (Bossuyt et al., 2026). Recent consensus work in China has further emphasized standardized syndrome classification, scoring rules, and efficacy evaluation methods for adult acute pharyngitis, which may improve comparability across future TCM studies (Yan-ning et al., 2025). In the present study, the TCM interventions under evaluation include marketed Chinese patent medicines, hospital-prepared formulations, individualized herbal prescriptions, and related therapeutic approaches (Liu et al., 2025; Fan et al., 2025; Lin et al., 2024b).

An increasing number of clinical studies and evidence reviews have examined traditional Chinese medicine (TCM) interventions for acute pharyngitis, including Chinese patent medicines, hospital-prepared formulations, compound herbal therapies, and individualized prescriptions (Fang et al., 2024; Ma et al., 2021). However, evidence synthesis remains limited by heterogeneity in outcome reporting, definition, and measurement, as well as by variation in syndrome differentiation, intervention strategies, comparator selection, and efficacy criteria across studies (Shi et al., 2007). This heterogeneity may be reflective of differences in the focus, scope, purpose, and setting of TCM interventions for acute pharyngitis, together with the tensions investigators face between comprehensive outcome assessment, study feasibility, and statistical power. Clinical recommendations are derived largely from evaluation of the medical literature, with randomized controlled trials (RCTs), particularly the synthesis of data from multiple RCTs, generally considered to provide the highest level of evidence (Braga et al., 2025). Such synthesis can, however, be difficult when similar therapeutic effects are assessed using non-uniform definitions, instruments, or reporting approaches, which in turn reduces comparability across studies and limits the interpretability of the available evidence (Zhang et al., 2023). In addition, outcome selection and evidence interpretation should not rely solely on the perspectives of clinicians and researchers, as patients, caregivers, and healthcare professionals may prioritize different outcomes and forms of evidence (Devane et al., 2025).

A Core Outcome Set (COS) represents an agreed minimum collection of outcomes to be measured and reported across all clinical trials in a defined healthcare area (Williamson et al., 2012). Beyond the minimum required outcomes, researchers may additionally measure and report other outcomes that are particularly pertinent to the scope of specific studies. Developing and applying COS within a particular clinical domain can reduce heterogeneity and bias in outcome reporting while also improving research efficiency (Williamson et al., 2017; Chalmers et al., 2014). The development of COS can enhance consistency and comparability among clinical trials, thereby increasing the value of trial findings for systematic reviews and meta-analyses, while also helping to reduce selective outcome reporting (Kirkham et al., 2017). The development of a COS generally follows a multistep process, beginning with a systematic or scoping review to identify outcomes reported in randomized controlled trials (RCTs), which provides an important basis for subsequent COS development (Williamson et al., 2017).

In 2023, a network meta-analysis coupled with data mining synthesized the published evidence on Chinese patent medicines used for acute pharyngitis (Keyan et al., 2023). Moreover, a Chinese-language systematic review published in 2017 evaluated traditional Chinese medicine interventions for acute pharyngitis (Mingsan et al., 2017). As COS development generally starts with identifying candidate outcomes for measurement, this review offers an important empirical starting point for future COS development in this area (Young et al., 2019). However, to our knowledge, no published review has systematically examined the broader spectrum of outcomes measured and reported in randomized controlled trials of traditional Chinese medicine for acute pharyngitis, nor the frequency with which these outcomes are reported; rather, existing reviews have primarily evaluated the efficacy and safety of particular interventions (Lin et al., 2024b; Ma et al., 2021). Therefore, the present scoping review was conducted as the first step toward developing a core outcome set for randomized controlled trials of traditional Chinese medicine for acute pharyngitis, with the aim of summarizing the outcomes reported in these trials (Williamson et al., 2017).

## Materials and methods

### Methodological framework

This scoping review was structured according to the foundational methodological framework proposed by Arksey and O’Malley (2005) and further informed by the Joanna Briggs Institute (JBI) guidelines. The process comprises five stages: (1) identifying the research question; (2) identifying relevant studies; (3) selecting eligible studies; (4) charting the data; and (5) collating, summarizing, and reporting the results. The optional sixth stage (consultation) was not undertaken in this review, as stakeholder engagement will be conducted during the subsequent Delphi survey and consensus process for core outcome set development. Finally, this review is reported in accordance with the PRISMA extension for Scoping Reviews (PRISMA-ScR).

### Registration

A systematic scoping review methodology was employed because it is particularly useful for systematically identifying and mapping the available evidence and for summarizing how research has been conducted in a given area (Munn et al., 2018). In addition, the Core Outcome Measures in Effectiveness Trials (COMET) Handbook recognizes scoping reviews as a valid approach for identifying existing outcome knowledge during COS development (Williamson et al., 2017).

This scoping review was registered with the COMET Initiative (registration number: 2,601). Established guidance for the conduct of scoping reviews was followed (Alexander et al., 2024), and reporting was undertaken in accordance with the PRISMA extension for Scoping Reviews (PRISMA-ScR) (Tricco et al., 2018). As this study involved the identification and synthesis of outcomes reported in published randomized controlled trials, rather than the collection of individual patient data, trial registry records, or other human participant data, institutional review board approval was not required.

### Search strategy

We systematically searched four English-language databases (PubMed, Web of Science, Embase, and the Cochrane Library) and four Chinese databases (CNKI, Wanfang Data, VIP, and SinoMed) from database inception to 30 September 2025. The search strategy combined controlled vocabulary terms, including MeSH and Emtree terms where applicable, with free-text terms for acute pharyngitis, traditional Chinese medicine, and randomized controlled trials (RCTs), using Boolean operators. The full PubMed search strategy is provided in Table 3 and was adapted for the other databases.

To identify prespecified outcomes, we additionally searched ChiCTR, ClinicalTrials.gov, and the WHO International Clinical Trials Registry Platform (ICTRP) for relevant trial registration records. Prespecified outcomes were extracted from eligible registration records and compared with the outcomes reported in the corresponding published articles.

### Study selection

All retrieved records were exported to Microsoft Excel for management and screening. Title and abstract screening and subsequent full-text assessment were independently conducted by two reviewers (Pang and Li) according to the predefined eligibility criteria. Disagreements were resolved through discussion and, when necessary, consultation with a third reviewer (Zhang). Records were retained for full-text assessment when eligibility could not be determined based on the title and abstract alone.

### Inclusion and exclusion criteria

The inclusion criteria for studies were:

- Any randomized controlled trial conducted in any country.
- Participants diagnosed with acute pharyngitis, regardless of age, sex, ethnicity, or care setting.
- Studies evaluating traditional Chinese medicine (TCM) interventions for acute pharyngitis, including Chinese patent medicines, hospital-prepared formulations, compound herbal medicines, individualized herbal prescriptions, and other oral or drug-based TCM therapies.
- Interventions delivered alone or in combination with conventional treatment, provided that the TCM component could be clearly identified.
- Studies comparing TCM interventions with placebo, no treatment, usual care, conventional medicine, or other active treatments.
- Trials reporting at least one clinical, symptomatic, laboratory, safety, or other outcome related to acute pharyngitis.
- Any duration of treatment or follow-up.
- For studies with duplicate or overlapping data, only the latest publication or the report providing the most complete information was retained.
- English language and Chinese language

The exclusion criteria for studies included:

- Non-randomized or quasi-randomized studies, observational studies, case series, case reports, reviews, editorials, letters, commentaries, study protocols, and conference abstracts without full-text data.
- Studies not specifically involving acute pharyngitis, or those including mixed upper respiratory conditions when data for acute pharyngitis could not be extracted separately.
- Studies focused on non-pharmacological traditional Chinese medicine interventions, such as acupuncture, moxibustion, cupping, massage, or acupoint stimulation.
- Studies in which Chinese medicine was combined with other treatments and the independent effect of the herbal intervention could not be determined.
- Studies evaluating prevention, rehabilitation, mechanistic exploration, pharmacology, animal experiments, or *in vitro* research rather than clinical treatment effects.
- Studies enrolling patients with other major throat-related diseases or specific infectious conditions that could confound the assessment of acute pharyngitis outcomes.
- Studies with unavailable, incomplete, or non-extractable outcome data.
- Articles published in languages other than Chinese or English.

### Data extraction

Following the recommendations of the COMET Initiative (Williamson et al., 2017), a Microsoft Excel–based data extraction form was developed. The first four included studies were used for pilot testing by two independent reviewers (Li and Pang), and discrepancies were checked through comparison. Once the extraction form was finalized, the same two reviewers independently recorded outcomes exactly as reported in the original articles in order to preserve transparency (Williamson et al., 2017). The extracted information comprised the study title, study start date, study completion date, recruitment status, study objectives and/or hypotheses, type of randomized controlled trial, country of recruitment, study setting, summary of the intervention, summary of the comparator, participant eligibility criteria, sample size, participant age, reported primary and secondary outcomes, outcome measurement instruments, outcome definitions, assessment time points, publication links, and details of the principal investigator and sponsor. When no link to a related publication was available, we searched for peer-reviewed articles using trial titles and keywords related to the lead author across the eight bibliographic databases described above. To ensure transparency, two independent reviewers additionally extracted verbatim any further relevant data from both linked and non-linked publications (Williamson et al., 2017).

### Data standardization

During outcome standardization, internationally and nationally recognized authoritative medical terminology standards were consulted, including SNOMED CT, MeSH, relevant clinical practice guidelines, the Chinese Traditional Medicine Subject Headings, and the National Standard of the People’s Republic of China—Clinical Terminology of Traditional Chinese Medicine Diagnosis and Treatment. For TCM-related outcomes, relevant national or industry standards were preferentially used to avoid ambiguity caused by non-standard terminology. Based on the expression and content of each outcome term, standardization was performed while preserving the original meaning. This process included correcting typographical errors, harmonizing synonyms and alternative names, standardizing English abbreviations, splitting or merging outcome terms where appropriate, clarifying specific referents, and combining identical outcomes under standardized labels (Table 4).

### Data analysis

In the present study, narrative synthesis was first applied to organise the included studies according to their design characteristics, reported outcome indicators, and measurement methods, while the COMET taxonomy served as the overarching framework for classification (Williamson et al., 2017). This approach was adopted to provide a transparent overview of outcome reporting and to reduce the influence of inconsistent or selective outcome reporting on subsequent evidence synthesis (Kirkham et al., 2010). Considering the distinctive features of traditional Chinese medicine, the outcome indicators were further refined and assigned to corresponding domains according to the seven-domain TCM classification framework developed by Professor Zhang Junhua and colleagues (Jianxin et al., 2021). In the classification stage, indicators specific to TCM were initially mapped onto the 12 COMET-recommended outcome categories and were subsequently organised by functional characteristics into seven domains: TCM disease and syndrome, symptoms/signs, physical and chemical examinations, quality of life, long-term prognosis, economic evaluation, and safety events (Jianxin et al., 2021; Williamson et al., 2017).

During the conceptualisation stage, two researchers worked collaboratively: Pang conducted the initial extraction and provisional classification of the indicators, while Li reviewed and merged indicators with similar definitions or conceptual overlap. The indicator domains and their definitions were then further revised by a multidisciplinary steering group, which included experts in COS development and senior scholars from relevant disciplines, through several rounds of consensus-based discussion until a final consensus was established (Kirkham et al., 2017). Statistically, the reporting frequency of each indicator was summarised using descriptive statistics, and phi coefficients combined with hierarchical clustering heatmaps were employed to further investigate the relationships among indicators and their distributional patterns.

Regarding the assessment of measurement properties, no quantitative appraisal of the measurement characteristics reported in the included studies was conducted at this review stage, as the objective of this review was to identify and classify outcomes rather than to select outcome measurement instruments (Gorst et al., 2020; Prinsen et al., 2016). Moreover, although COSMIN offers methodological guidance for evaluating PROMs (Mokkink et al., 2018), no universally accepted appraisal framework is currently available for all TCM-specific outcome measures, particularly syndrome-related indicators (Zonghai et al., 2022).

## Results

### Search results and study selection

A total of 6,235 records were identified through database searching. After the removal of 2,240 duplicate records, 3,995 records were retained for title and abstract screening. Of these, 2,248 records were excluded because they did not meet the eligibility criteria. The remaining 1,747 full-text articles were assessed for eligibility. During the full-text review, 1,565 articles were excluded for the following reasons: ineligible population (n = 1,204), ineligible intervention (n = 168), ineligible study design (n = 178), non-extractable outcome data (n = 5), duplicate or overlapping data (n = 7), and unavailable full text (n = 3).

**FIGURE 1:**
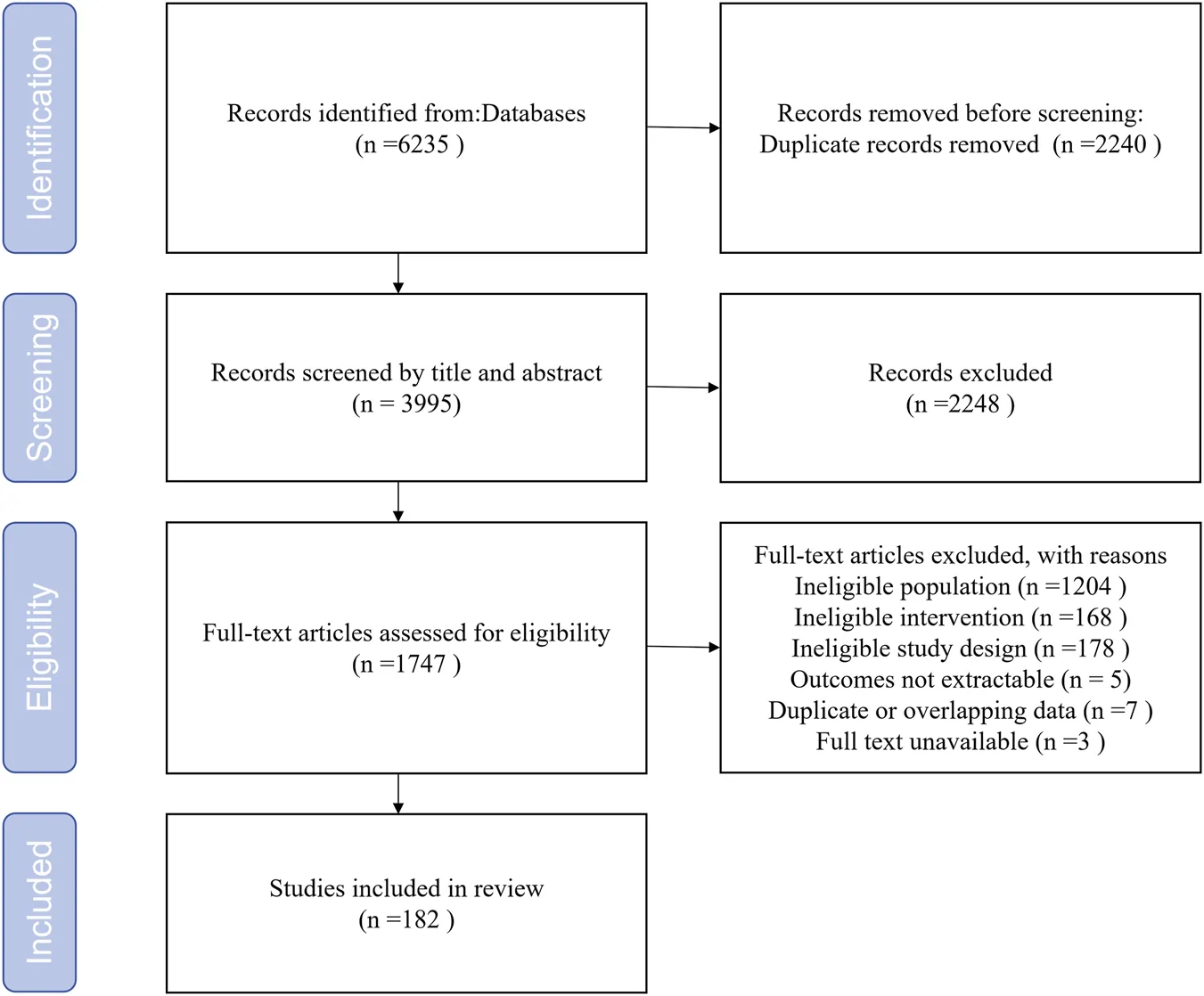
PRISMA diagram of study selection for inclusion into the review.

Specifically, excluded populations mainly involved patients with other respiratory or throat conditions that could not be clearly separated from pharyngitis. Ineligible interventions primarily consisted of external TCM therapies outside our predefined scope of oral TCM treatments. Additionally, studies with ineligible designs were largely non-randomized trials or animal experiments. Through this rigorous screening process, a highly relevant subset of 182 studies strictly meeting the inclusion criteria was finally identified and included in the review. Details of the study selection process are presented in the PRISMA flow diagram (Figure 1).

### Study characteristics

The 182 included studies enrolled a total of 25,230 participants. Across studies, the sample size varied from 40 to 756 participants, and the median sample size was 105 participants (IQR, 78–160). Based on the intervention forms described in the included studies, we grouped the TCM interventions into six categories for descriptive analysis. Marketed Chinese patent medicines (standardized, commercially manufactured herbal formulations approved for clinical use by regulatory authorities) were the most frequently investigated intervention type, accounting for 133 studies (73.1%), followed by individualized prescriptions (herbal formulas tailored to individual patients based on traditional syndrome differentiation principles) in 26 studies (14.3%) and hospital preparations (in-house herbal formulations prepared and dispensed within specific medical institutions for internal clinical use) in 15 studies (8.2%). Empirical formulas (fixed herbal prescriptions developed based on clinicians’ experience rather than individualized modification) were evaluated in four studies (2.2%), whereas investigational new Chinese medicines (novel herbal products under clinical investigation that have not yet received regulatory approval for routine clinical use) and combined regimens were each reported in two studies (1.1%).

**FIGURE 2:**
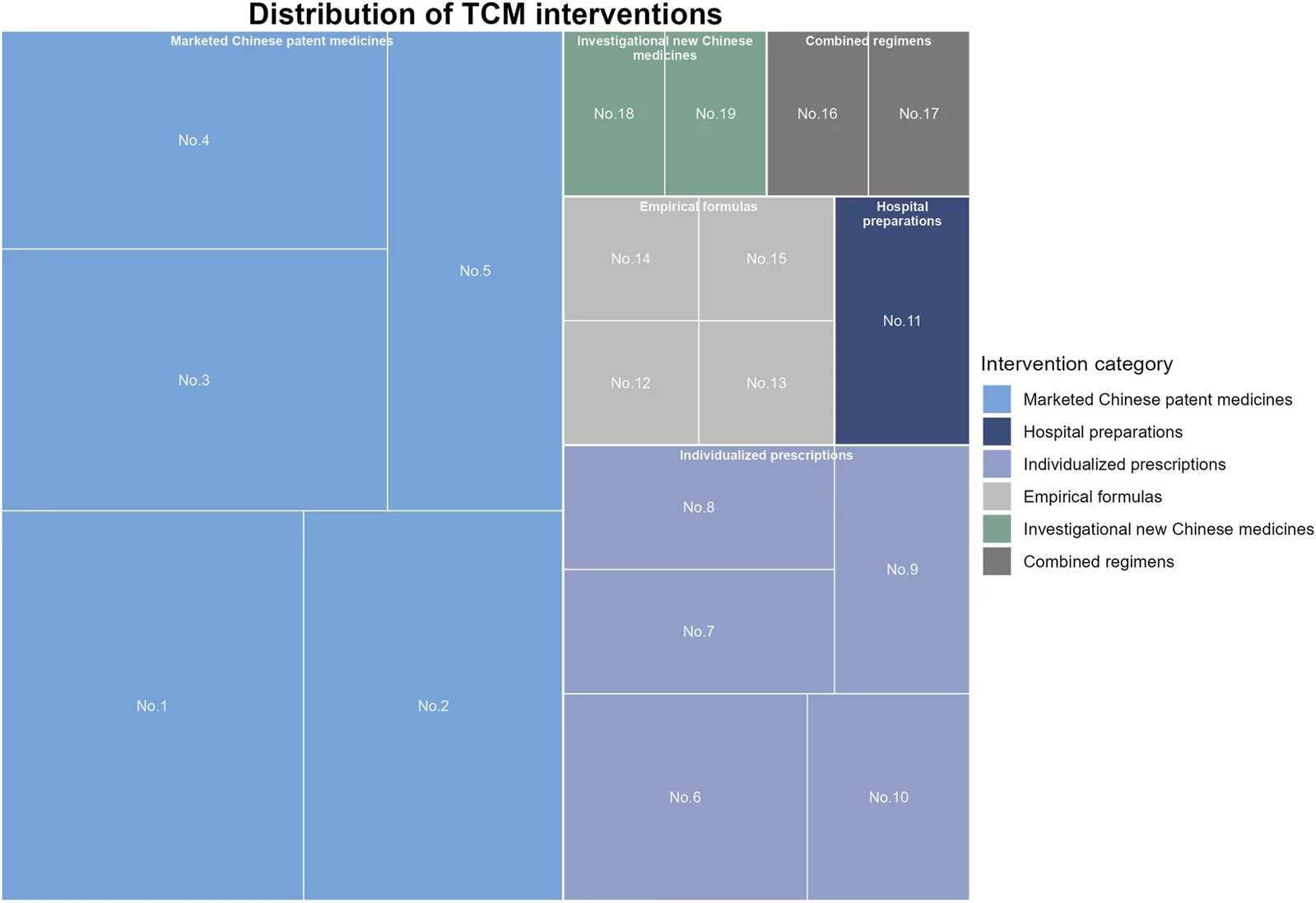
Hierarchical treemap of the five most frequently reported specific interventions within each category.

To provide a clearer overview of the distribution of interventions, Figure 2 presents a hierarchical treemap of the five most frequently reported specific interventions within each category. Among marketed Chinese patent medicines, the most commonly reported were Shufeng Jiedu capsules (7, 3.8%), Ertong Qingyan Jiere oral liquid (6, 3.3%), Lanqin oral liquid (6, 3.3%), Ganjie Bingmei tablets (5, 2.7%), and Qingyan dropping pills (5, 2.7%). Within the category of individualized prescriptions, Qingrun decoction (3, 1.6%) was the most frequently reported, followed by modified Gancao-Jiegeng-Shegan decoction (2, 1.1%), modified Shufeng Qingre decoction (2, 1.1%), modified Qingyan Chubi decoction (2, 1.1%), and Qingyan Lige formula granules (2, 1.1%). For hospital preparations, Yanhouqing lozenges (2, 1.1%) represented the leading intervention. Empirical formulas included Chuihou powder (1, 0.5%), Huanglian Qing Drink Soup (1, 0.5%), Yinshe lozenges (1, 0.5%), and Zizheng Dihuang decoction (1, 0.5%). Combined regimens comprised Kangfuxin combined with Dual-material Houfeng Powder (1, 0.5%) and Yinqiao Powder combined with Qingkailing injection (1, 0.5%). Investigational new Chinese medicines included artificial bear bile powder capsules (1, 0.5%) and Banzhilian total flavonoids capsules (1, 0.5%). The detailed compositions of these multi-component preparations are provided in Supplementary Data Sheet 1.

**FIGURE 3:**
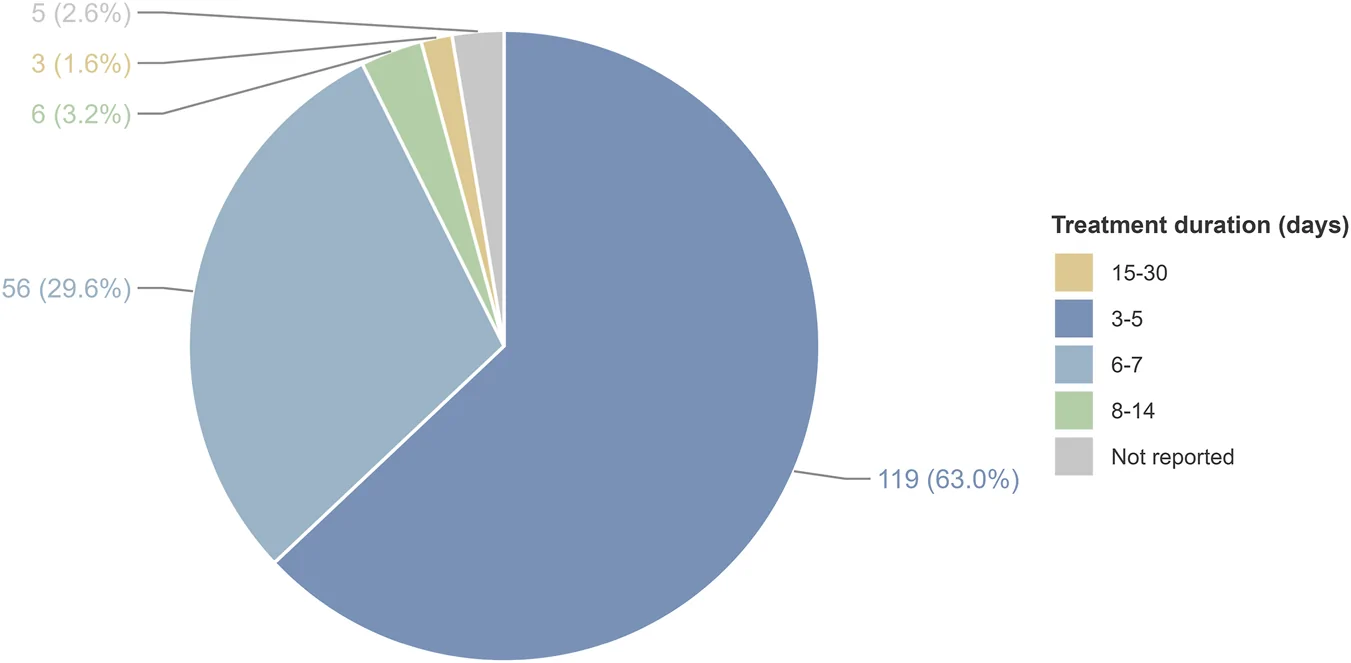
Distribution of treatment duration among the included studies.

As shown in Figure 3, treatment duration was generally short among the included studies. The most frequently reported treatment course was 3–5 days, which was used in 119 studies (63.0%), followed by 6–7 days in 56 studies (29.6%). Longer treatment durations were relatively uncommon, with 8–14 days reported in six studies (3.2%) and 15–30 days in three studies (1.6%). Five studies (2.6%) did not provide information on treatment duration. Overall, the majority of TCM interventions were administered within 1 week.

Intervention codes: No.1, Shufeng Jiedu capsules; No.2, Ertong Qingyan Jiere oral liquid; No.3, Lanqin oral liquid; No.4, Ganjie Bingmei tablets; No.5, Qingyan dropping pills; No.6, Qingrun decoction; No.7, modified Gancao-Jiegeng-Shegan decoction; No.8, modified Shufeng Qingre decoction; No.9, modified Qingyan Chubi decoction; No.10, Qingyan Lige formula granules; No.11, Yanhouqing lozenges; No.12, Chuihou powder; No.13, Huanglian Qing Drink Soup; No.14, Yinshe lozenges; No.15, Zizheng Dihuang Decoction; No.16, Kangfuxin + Dual material Houfeng Powder; No.17, Yinqiao Powder + Qingkailing Injection; No.18, Artificial bear bile powder capsules; No.19, Banzhilian Total Flavonoids Capsules.

### Outcome domains

Based on the seven-domain TCM classification framework (Jianxin et al., 2021), 817 individual outcomes were extracted from the 182 studies included in this review and classified into six broad domains, as summarized in Table 1. No eligible outcomes could be mapped to the long-term prognosis domain; this domain was retained in the framework to preserve its completeness and to highlight this evidence gap. Ten eligible trial registration records were identified. All prespecified outcomes in these records were reported in the corresponding published articles; therefore, extracting outcomes from trial registries did not change the candidate outcome list, which remained at 817 individual outcomes. Some published articles also reported additional outcomes beyond those prespecified in the corresponding registration records.

**TABLE 1:** Outcome domains.

Outcome domain	Outcome	Frequency [times (%)]
Symptoms/Signs	Clinical efficacy	168 (29.0%)
	Sore throat	77 (13.3%)
	Fever	44 (7.6%)
	Pharyngeal congestion	35 (6.0%)
	Dry and burning throat	26 (4.5%)
	Cough	23 (4.0%)
	Pharyngeal redness and swelling	17 (2.9%)
	Dry throat	16 (2.8%)
	Thirst	15 (2.6%)
	VAS	10 (1.7%)
	Foreign body sensation in the throat	10 (1.7%)
	Dry stool	9 (1.6%)
	Difficulty swallowing	9 (1.6%)
	Yellow urine	9 (1.6%)
	Coughing and expectoration	8 (1.4%)
	Abnormal tongue image	6 (1.0%)
	Headache	6 (1.0%)
	Itchy throat	6 (1.0%)
	Hoarseness	5 (0.9%)
	Symptoms	5 (0.9%)
	Enlargement of submandibular lymph nodes	4 (0.7%)
	Abnormal pulse condition	4 (0.7%)
	Uvula edema	4 (0.7%)
	Redness and swelling of the lateral pharyngeal cord	4 (0.7%)
	Redness and swelling of follicles on the posterior pharyngeal wall	4 (0.7%)
	Main symptoms	4 (0.7%)
	Secondary symptoms	3 (0.5%)
	Aversion to cold	3 (0.5%)
	Expectoration	3 (0.5%)
	Pharyngeal examination	3 (0.5%)
	Astriction	2 (0.3%)
	Decreased secretion	2 (0.3%)
	Tenderness of submandibular lymph nodes	2 (0.3%)
	Halitosis	2 (0.3%)
	Oral herpes	2 (0.3%)
​	Lymph node enlargement	2 (0.3%)
	Edema of soft palate and velar	2 (0.3%)
	Thick phlegm	2 (0.3%)
	Swelling and swelling of the uvula	2 (0.3%)
	Pharyngeal signs	2 (0.3%)
	Redness and swelling of pharyngeal mucosa/uvula	2 (0.3%)
	Symptoms and signs	2 (0.3%)
	EAT10 rating	1 (0.2%)
	Enlarged tonsils	1 (0.2%)
	Swelling and pain of submandibular lymph nodes	1 (0.2%)
	Results of laryngoscopy examination	1 (0.2%)
	Mental restlessness or fatigue	1 (0.2%)
	Degree of damage to oropharyngeal mucosa	1 (0.2%)
	Abnormal pulse and fingerprint	1 (0.2%)
	Hot eyes	1 (0.2%)
	Pus plug	1 (0.2%)
	Systemic symptoms	1 (0.2%)
	Loss of appetite	1 (0.2%)
	Headache and body aches	1 (0.2%)
	Discomfort in the throat	1 (0.2%)
	Pharyngeal lymphoid follicles	1 (0.2%)
	Dry throat and sore throat	1 (0.2%)
Quality of life	BPI rating	1 (12.5%)
	Mood	1 (12.5%)
	Daily work	1 (12.5%)
	Daily life	1 (12.5%)
	Life interests	1 (12.5%)
	Slumber	1 (12.5%)
	Walking ability	1 (12.5%)
	Relationship with others	1 (12.5%)
Physical and chemical examination	TNF-α	20 (12.1%)
	IL-6	17 (10.3%)
	WBC	11 (6.7%)
	CRP	10 (6.1%)
	IL-1β	9 (5.5%)
	CD4^+^	8 (4.8%)
	hs-CRP	7 (4.2%)
	CD3^+^	6 (3.6%)
	CD8^+^	6 (3.6%)
	SlgA	6 (3.6%)
	CD4+/CD8+	5 (3.0%)
	IL-2	5 (3.0%)
	Pharynx swab culture	4 (2.4%)
	Neutrophil	4 (2.4%)
	IL-8	2 (1.2%)
	Th1	2 (1.2%)
	Th1/Th2	2 (1.2%)
	INF-γ	2 (1.2%)
	ALT	2 (1.2%)
	RBC	2 (1.2%)
	GR	2 (1.2%)
	LY	2 (1.2%)
	ECG	2 (1.2%)
	VCAM-1	2 (1.2%)
	Hb	2 (1.2%)
	Cr	2 (1.2%)
	BUN	2 (1.2%)
	IL-1	1 (0.6%)
	IL-4	1 (0.6%)
	IL-5	1 (0.6%)
	Th2	1 (0.6%)
	γ-GT	1 (0.6%)
	AST	1 (0.6%)
	MMP-9	1 (0.6%)
	ALP	1 (0.6%)
	PCT	1 (0.6%)
	FPG	1 (0.6%)
	LEU	1 (0.6%)
​	Urine Routine Examination	1 (0.6%)
	PRO	1 (0.6%)
	GLU	1 (0.6%)
	BLD	1 (0.6%)
	Lysozyme	1 (0.6%)
	Abnormal laboratory examination	1 (0.6%)
	EOS	1 (0.6%)
	SRT	1 (0.6%)
	PLT	1 (0.6%)
	TBIL	1 (0.6%)
Economic evaluation	Time to recovery	10 (52.6%)
	Cost-effectiveness ratio (C-E)	1 (5.3%)
	Indirect cost	1 (5.3%)
	Onset time	1 (5.3%)
	Cost of experimental drugs	1 (5.3%)
	Medical cost	1 (5.3%)
	Incremental cost-effectiveness ratio	1 (5.3%)
	Direct non-medical costs	1 (5.3%)
	Total direct medical costs	1 (5.3%)
	Total cost	1 (5.3%)
TCM Syndrome	TCM Syndrome Efficacy	16 (45.7%)
	TCM Syndrome Score	13 (37.1%)
	Wind-heat syndrome	3 (8.6%)
	Lung stomach heat syndrome	2 (5.7%)
	Spleen and stomach damp heat syndrome	1 (2.9%)
Security event	Adverse event	1 (100%)

The most frequently reported outcome domain was Symptoms/Signs (n = 182, 100%), reflecting the central role of symptom assessment in acute pharyngitis trials. All included studies reported at least one outcome within this domain. Physical and chemical examination was the second most commonly reported domain (n = 47, 25.8%), followed by TCM Syndrome (n = 34, 18.7%). Security event outcomes were reported in 15 studies (8.2%), while Economic evaluation was addressed in nine studies (4.9%). Quality of life was the least frequently reported domain, identified in only two studies (1.1%), despite multiple related measures being reported within individual trials (Table 1). The mean number of outcome domains per study was 1.62 (range 1–4; median = 1, IQR = 1). The study reporting the widest range of outcome domains included four domains.

**FIGURE 4:**
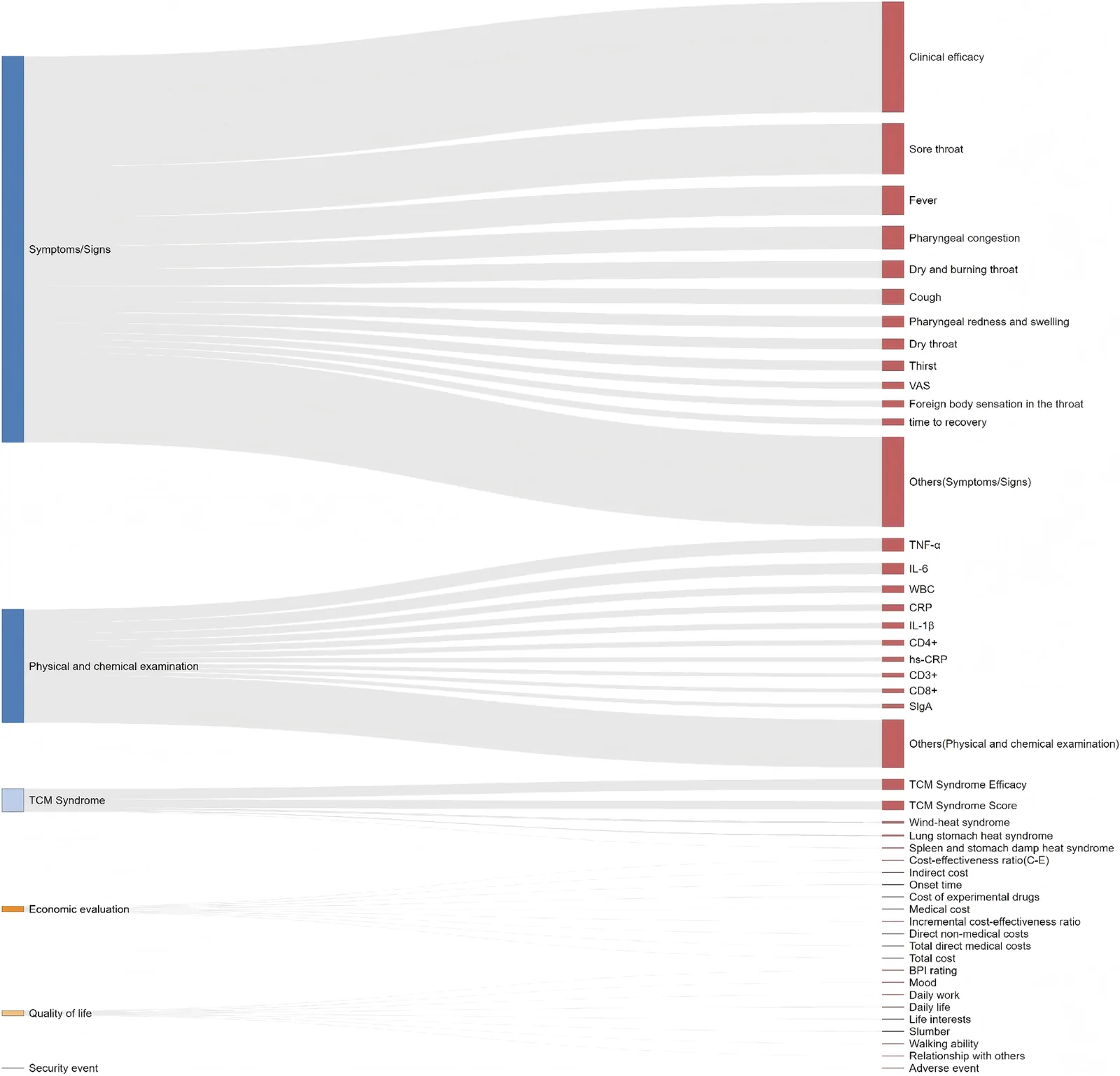
Sankey diagram illustrating the distribution of outcomes across the six predefined domains.

Figure 4 presents the distribution of outcomes across the six predefined domains using a Sankey diagram. Overall, outcomes were predominantly concentrated in the Symptoms/Signs domain, indicating that clinical studies mainly focused on symptom relief and overall therapeutic response. Within this domain, clinical efficacy was the most frequently reported outcome (168,20.6%), followed by sore throat (77,9.4%), fever (44,5.4%), pharyngeal congestion (35,4.3%), dry and burning throat (26,3.2%), cough (23,2.8%), pharyngeal redness and swelling (17,2.1%), dry throat (16,2.0%), thirst (15,1.8%), VAS (10,1.2%), time to recovery (10,1.2%) and foreign body sensation in the throat (10,1.2%). Outcomes classified as others within this domain also accounted for a substantial proportion (137,16.8%), reflecting considerable heterogeneity in symptom-related outcome selection. The second largest domain was Physical and chemical examination, in which inflammatory and immune-related indicators were commonly used, including TNF-α (20,2.4%), IL-6 (17,2.1%), WBC (11,1.3%), CRP (10,1.2%), IL-1β (9,1.1%), CD4^+^ (8,1.0%), hs-CRP (7,0.9%), CD3^+^ (6,0.8%), CD8^+^ (6,0.8%), and SIgA (6,0.8%), with an additional 73 (8.9%) outcomes grouped as others. In the TCM Syndrome domain, TCM syndrome efficacy (16,2.0%) and TCM syndrome score (13,1.6%) were reported most often, whereas specific syndrome types, such as wind-heat syndrome (3,0.3%), lung-stomach heat syndrome (2,0.2%), and spleen-stomach damp-heat syndrome (1,0.1%), were reported less frequently. Outcomes related to Economic evaluation, Quality of life, and Security event were relatively scarce. The Economic evaluation domain contained only a small number of cost- or cost-effectiveness-related outcomes, all of which were reported once. Quality-of-life outcomes, all derived from the Brief Pain Inventory (BPI), included the BPI rating and interference-related items such as mood, daily work, daily life, life interests, slumber, walking ability, and relationship with others; each item was reported once. Only one outcome, adverse event, was identified in the Security event domain. This distribution pattern revealed a clear imbalance in outcome selection in current pharyngitis trials, with a strong emphasis on acute and subjective symptom assessments rather than multidimensional or long-term evaluations. Although objective inflammatory and immune biomarkers were also reported, their reporting frequencies were substantially lower than those of symptom-related outcomes. Moreover, these domains were generally assessed separately rather than being systematically integrated, reflecting a fragmented approach to clinical evaluation.

**FIGURE 5:**
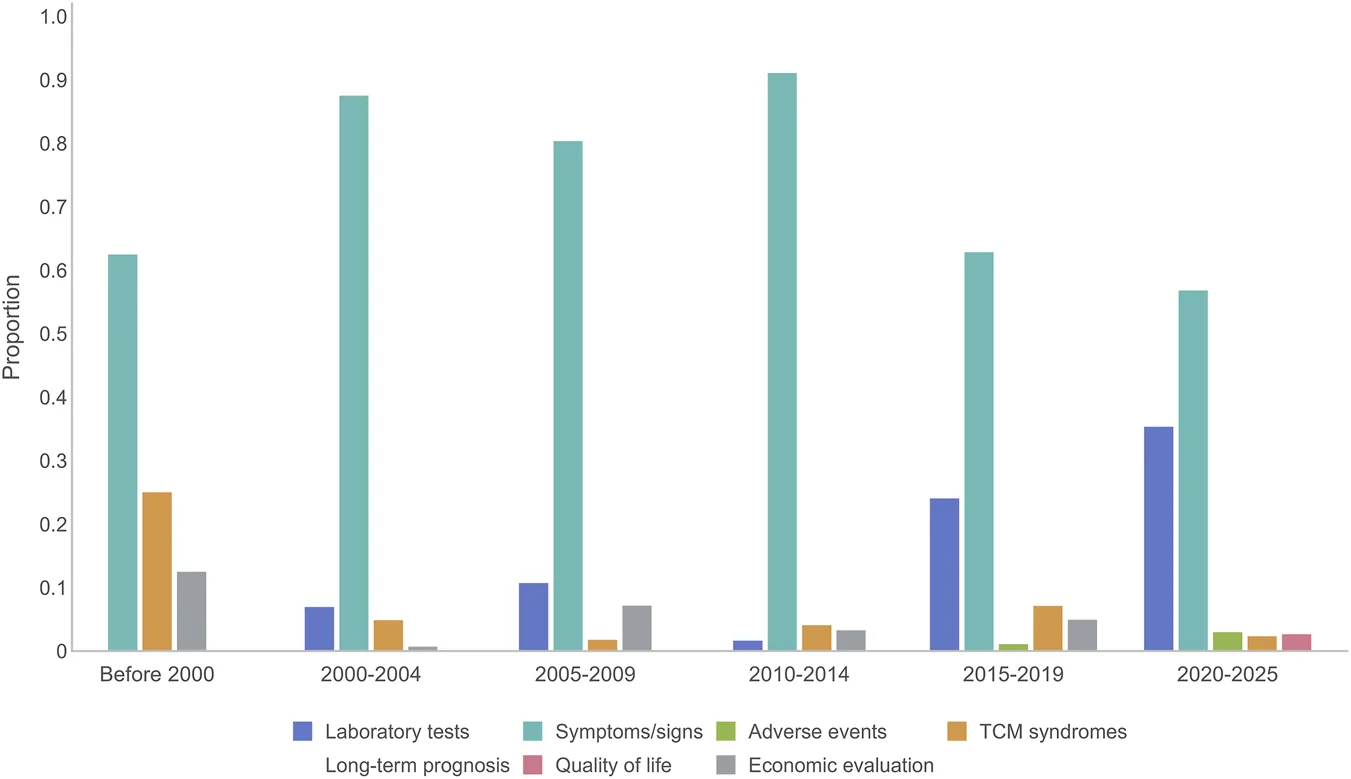
Temporal trends in the distribution of outcome domains across included studies.

Temporal analysis demonstrated a clear shift in the distribution of outcome domains across publication periods (Figure 5). Symptoms/Signs consistently represented the predominant domain throughout all time intervals, with proportions remaining above 60% and peaking at over 90% during 2010–2014. In contrast, Physical and chemical examination outcomes showed a gradual increase over time, rising markedly after 2015 and reaching their highest level in 2020–2025. TCM Syndrome-related outcomes were more frequently reported in earlier periods, particularly before 2000, but their relative contribution declined in subsequent years despite a transient increase between 2015 and 2019. Economic evaluation outcomes were sporadically reported, with a modest presence in earlier and mid-period studies, followed by an absence in the most recent period. Security event outcomes emerged only in later years, with a slight increase after 2015. Notably, Quality of life outcomes were absent in studies published before 2020 but appeared in the most recent period, indicating a growing emphasis on patient-centered measures. No studies across any time period reported outcomes related to long-term prognosis. Overall, these findings suggest a transition from a symptom-dominated evaluation framework toward a more diversified outcome structure incorporating laboratory indicators, safety, and patient-reported outcomes.

### Outcomes

Among the 182 included studies, clinical efficacy emerged as the primary outcome (84.6%, n = 154). Symptom-based assessments predominantly focused on sore throat (n = 120, 65.9%), fever (n = 68, 37.4%), and pharyngeal congestion (n = 62, 34.1%). Assessments of cough and pharyngeal irritation (dryness/burning) were moderately represented, occurring in 30.2% and 26.4% of trials, respectively. Less common outcomes included dysphagia, pharyngeal paresthesia, and hoarseness, all reported in distal proportions (<12%). Notably, the adoption of standardized patient-reported measures (e.g., VAS) remained limited (8.2%), with most other specific outcomes falling below a 5% reporting threshold. This pattern suggests that existing trials have primarily evaluated TCM interventions for pharyngitis through short-term symptomatic relief and overall treatment response.

**FIGURE 6:**
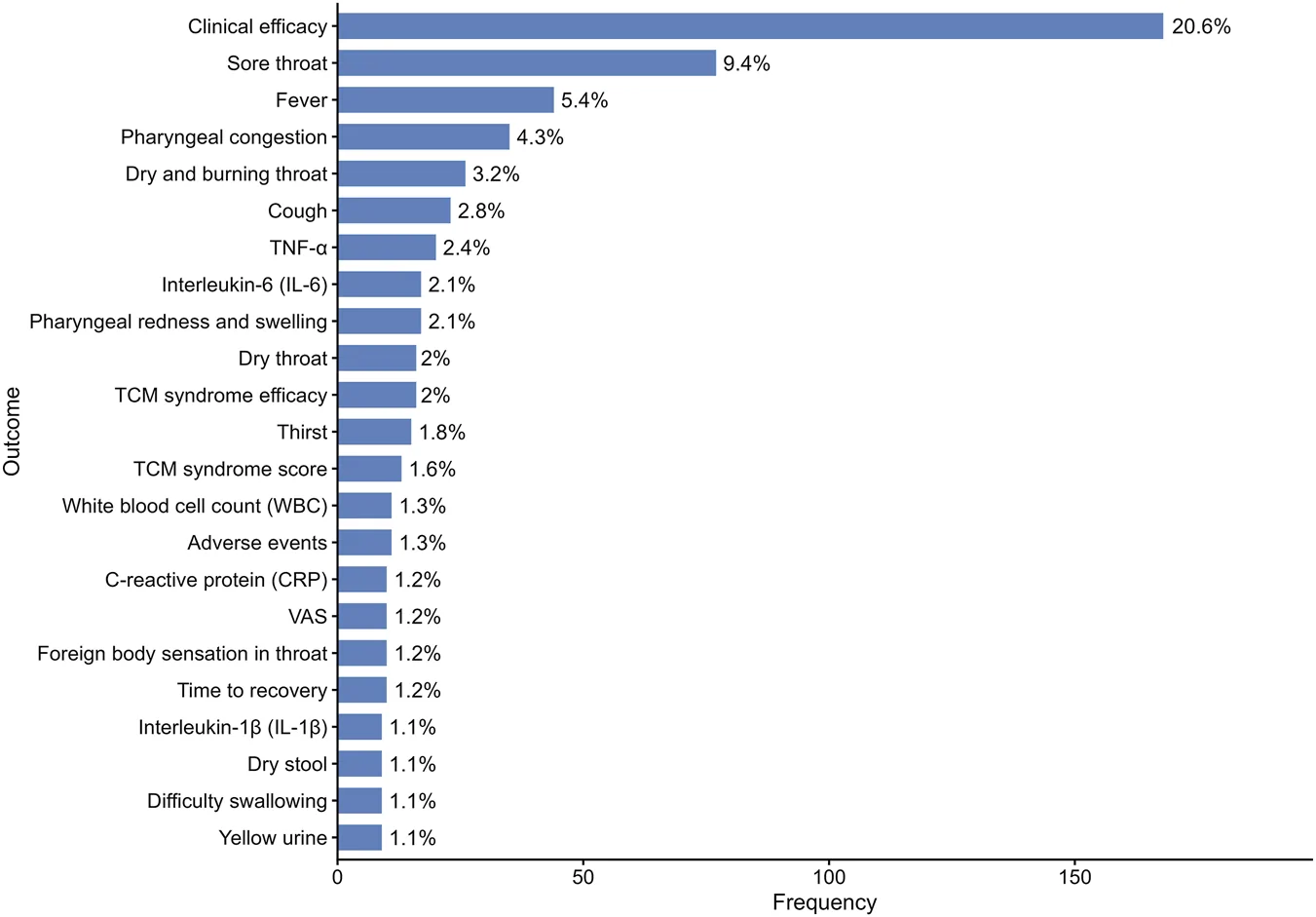
Bar chart of the 20 most frequently reported outcome measures across included studies.

Analysis of outcome frequency revealed that clinical efficacy was the most frequently reported indicator (Figure 6), accounting for 20.6% of all outcome occurrences (n = 168). This was followed by several symptom-related outcomes, including sore throat (n = 77, 9.4%), fever (n = 44, 5.4%), and pharyngeal congestion (n = 35, 4.3%). Other commonly reported symptoms comprised dry and burning throat (n = 26, 3.2%) and cough (n = 23, 2.8%). In addition, a number of inflammatory and laboratory markers were among the top-ranked outcomes, such as TNF-α (n = 20, 2.4%), interleukin-6 (IL-6) (n = 17, 2.1%), white blood cell count (WBC) (n = 11, 1.3%), and C-reactive protein (CRP) (n = 10, 1.2%). TCM-related measures, including TCM syndrome efficacy (n = 16, 2.0%) and TCM syndrome score (n = 13, 1.6%), were also frequently reported. Other outcomes, such as pharyngeal redness and swelling (n = 17, 2.1%), dry throat (n = 16, 2.0%), thirst (n = 15, 1.8%), adverse events (n = 11, 1.3%), visual analogue scale (VAS) scores (n = 10, 1.2%), foreign body sensation in the throat (n = 10, 1.2%), time to recovery (n = 10, 1.2%), and interleukin-1β (IL-1β) (n = 9, 1.1%) were reported with moderate to low frequency. Several additional outcomes, including dry stool, difficulty swallowing, and yellow urine (each n = 9, 1.1%), were among the least frequently reported within the top-ranked indicators. Overall, the distribution of the most commonly reported outcomes highlights a predominance of symptom-based measures, supplemented by selected laboratory, TCM-specific, and safety-related indicators.These estimates are derived from outcome-level frequencies and therefore differ from study-level reporting proportions presented above.

**FIGURE 7:**
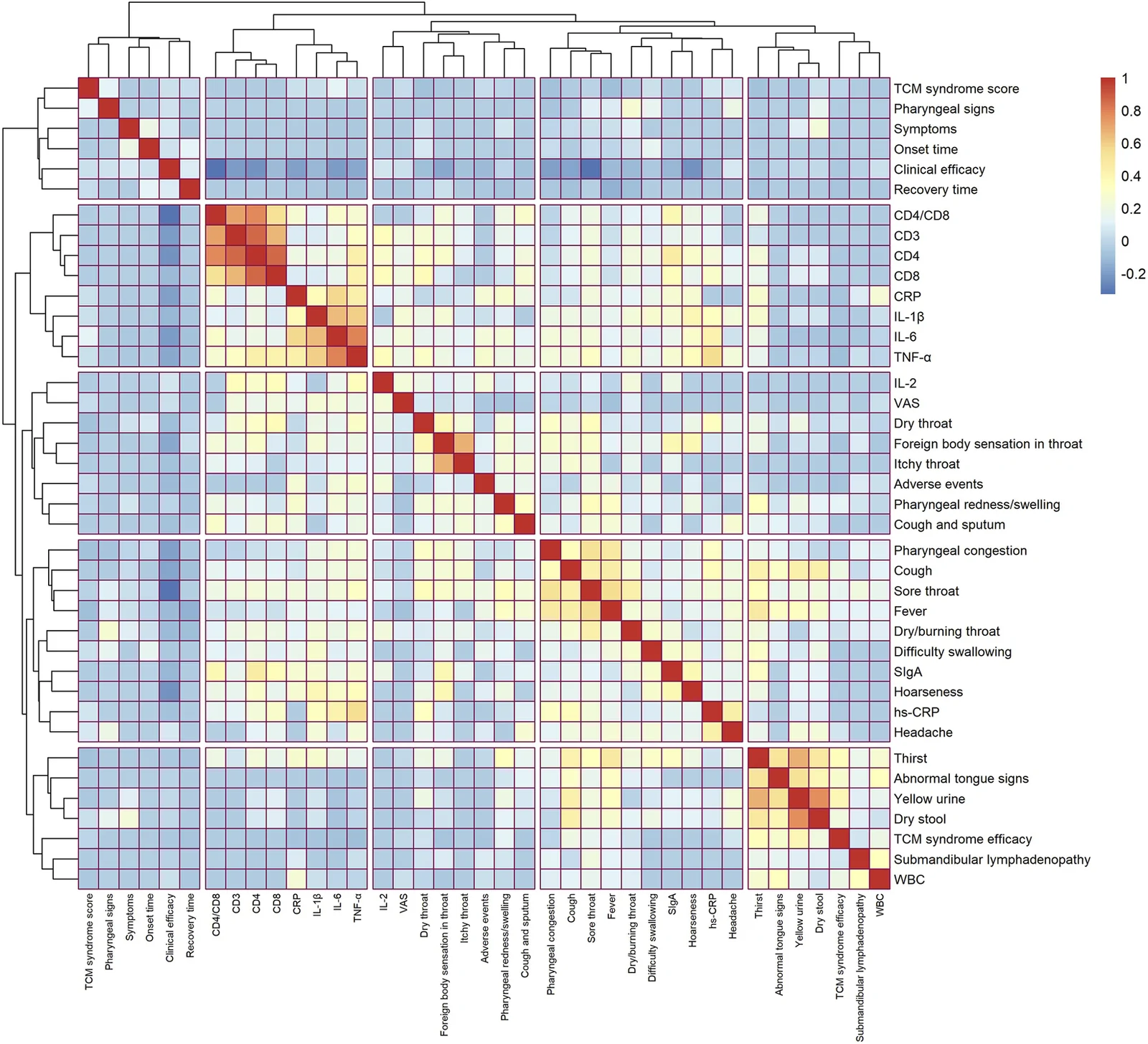
Phi coefficient matrix with hierarchical clustering of outcome measures.

The Phi coefficient matrix combined with hierarchical clustering revealed distinct patterns of co-occurrence among outcome measures (Figure 7). Strong positive associations were observed among inflammatory biomarkers, particularly between IL-6 and TNF-α (φ = 0.80), as well as between IL-6 and IL-1β (φ = 0.65), indicating that these markers were frequently assessed together within the same studies. Similarly, immune-related indicators formed a tightly connected cluster, with high correlations between CD3^+^ and CD4^+^ (φ = 0.86), CD4^+^ and CD8^+^ (φ = 0.86), and CD4^+^/CD8^+^ ratio with CD4^+^ (φ = 0.78), suggesting a consistent pattern of combined immunological evaluation.Symptom-related outcomes also demonstrated moderate clustering. For example, sore throat showed notable associations with fever (φ = 0.56) and pharyngeal congestion (φ = 0.53), while cough was moderately correlated with both fever and throat-related discomfort (φ ≈ 0.44–0.45). In addition, TCM-related manifestations, such as thirst, yellow urine, and dry stool, exhibited relatively strong internal correlations (φ up to 0.77), reflecting their tendency to be reported collectively within syndrome-based assessments.In contrast, clinical efficacy and time-to-event outcomes (e.g., time to recovery, onset time) showed weak or negligible correlations with most other indicators, suggesting that these measures were often reported independently rather than as part of a structured outcome set. Adverse events and patient-reported outcomes (e.g., VAS) also displayed generally low correlation coefficients, indicating limited integration with other outcome domains.Overall, the clustering structure highlights a fragmented outcome reporting pattern, characterized by tightly grouped biomarker and immunological measures alongside more loosely connected symptom and clinical endpoints.

### Heterogeneity of outcome reporting

Marked heterogeneity was identified in the measurement approaches applied to frequently reported outcomes (Table 2). In this analysis, measurement methods and judgment criteria were examined for outcomes with a reporting frequency greater than 10. Among these outcomes, the majority were assessed using two or more measurement tools or evaluation standards. For instance, clinical efficacy—the most frequently reported endpoint—was evaluated using 20 distinct criteria, primarily derived from national-level guidelines (e.g., Guiding Principles for Clinical Research of New Chinese Medicines), professional society standards, specialty textbooks, and, in some cases, study-specific definitions. A similar pattern was observed for symptom-based outcomes, including sore throat, fever, and pharyngeal congestion, which were measured using a wide array of sources, ranging from formal diagnostic criteria to narrative clinical descriptions, with only limited use of standardized instruments such as the visual analogue scale (VAS).In contrast, laboratory-based outcomes, such as tumor necrosis factor-α (TNF-α) and interleukin-6 (IL-6), demonstrated substantially greater consistency, with measurements predominantly conducted using enzyme-linked immunosorbent assay (ELISA). This contrast underscores a clear distinction between subjective symptom-based assessments and objective biochemical indicators, with the latter benefiting from more standardized and reproducible measurement techniques.Notably, incomplete reporting of measurement methods remained a concern. For clinical efficacy, 52 out of 207 studies (25.1%) did not specify the criteria used, while similar issues were observed for outcomes such as white blood cell count (WBC) and adverse events, where reporting of measurement details was frequently absent or insufficient.

**TABLE 2:** Measurement tools and judgment criteria for outcomes with a reporting frequency greater than 10.

Frequent outcomes	Number of studies reporting this outcome measure	Number of types of measuring tools/judgment criteria	Measuring tools/judgment criteria (Number)	Number of studies that did not report measuring tools/judgment criteria
Clinical efficacy	207	20	“Guiding Principles for Clinical Research of New Chinese Medicines” (104); “Criteria for Diagnosis and Therapeutic Effect of Diseases and Syndromes in Traditional Chinese Medicine” (31); “Otorhinolaryngology in Traditional Chinese Medicine” (7); “Criteria for Clinical Diagnosis and Standards for Cure and Improvement” (4); “Practical Otorhinolaryngology” (4); “Otorhinolaryngology” (4); “Practical Internal Medicine” (4); “Zhu Futang’s Practical Pediatrics” (3); “Diagnostic Otorhinolaryngology” (3); “Pediatrics” (1); “Otorhinolaryngology–Head and Neck Surgery” (1); “Criteria for Clinical Disease Diagnosis and Evaluation of Therapeutic Effect” (1); “Practical Neonatology” (1); “Modern Diagnosis and Treatment in Pediatric Traditional Chinese Medicine” (1); “Integrative Chinese and Western Otorhinolaryngology” (1); “Routine Diagnosis and Treatment of Diseases and Syndromes in Traditional Chinese Medicine” (1); “Pediatrics of Traditional Chinese Medicine” (1); “Guidelines for Diagnosis and Treatment of Common Otorhinolaryngologic Diseases in Traditional Chinese Medicine” (1); “Guidelines for Diagnosis and Treatment of Otorhinolaryngology–Head and Neck Surgery Diseases” (1); “Guidelines for Diagnosis and Treatment of Common Diseases in Internal Medicine: TCM Disease and Syndrome Section” (1)	52
Sore throat	95	18	“Guiding Principles for Clinical Research of New Chinese Medicines” (46); “Criteria for Diagnosis and Therapeutic Effect of Diseases and Syndromes in Traditional Chinese Medicine” (14); “Otorhinolaryngology in Traditional Chinese Medicine” (11); Visual analogue scale (VAS) (8); “Practical Otorhinolaryngology” (2); “Zhu Futang’s Practical Pediatrics” (2); “Diagnostic Otorhinolaryngology” (2); “Practical Internal Medicine” (2); “Otorhinolaryngology” (1); “Otorhinolaryngology–Head and Neck Surgery” (1); “Clinical Otorhinolaryngology–Head and Neck Surgery” (1); “Practical Otorhinolaryngology–Head and Neck Surgery” (1); “Modern Diagnosis and Treatment in Pediatric Traditional Chinese Medicine” (1); “Introduction to the Four-Grade Weighted Scoring Method for Clinical Diagnosis and Evaluation of Therapeutic Effect” (1); “Pediatrics of Traditional Chinese Medicine” (1); “Criteria for Diagnosis and Therapeutic Effect of Otorhinolaryngologic Diseases and Syndromes in Traditional Chinese Medicine” (1); “Guidelines for Diagnosis and Treatment of Common Otorhinolaryngologic Diseases in Traditional Chinese Medicine” (1); “Guidelines for Diagnosis and Treatment of Otorhinolaryngology–Head and Neck Surgery Diseases” (1)	15
Fever	62	15	“Guiding Principles for Clinical Research of New Chinese Medicines” (20); “Criteria for Diagnosis and Therapeutic Effect of Diseases and Syndromes in Traditional Chinese Medicine” (11); “Diagnostic Otorhinolaryngology” (7); “Otorhinolaryngology in Traditional Chinese Medicine” (5); “Practical Otorhinolaryngology–Head and Neck Surgery” (2); “Guidelines for Diagnosis and Treatment of Otorhinolaryngology–Head and Neck Surgery Diseases” (2); “Practical Internal Medicine” (2); “Pediatrics” (1); “Clinical Otorhinolaryngology–Head and Neck Surgery” (1); “Criteria for Clinical Diagnosis and Standards for Cure and Improvement” (1); “Practical Otorhinolaryngology” (1); “Modern Diagnosis and Treatment in Pediatric Traditional Chinese Medicine” (1); “Introduction to the Four-Grade Weighted Scoring Method for Clinical Diagnosis and Evaluation of Therapeutic Effect” (1); “Pediatrics of Traditional Chinese Medicine” (1); “Zhu Futang’s Practical Pediatrics” (1)	19
Pharyngeal congestion	38	12	“Guiding Principles for Clinical Research of New Chinese Medicines” (15); “Criteria for Diagnosis and Therapeutic Effect of Diseases and Syndromes in Traditional Chinese Medicine” (12); Visual analogue scale (VAS) (3); “Otorhinolaryngology in Traditional Chinese Medicine” (3); “Otorhinolaryngology–Head and Neck Surgery” (1); “Clinical Otorhinolaryngology–Head and Neck Surgery” (1); “Clinical Practice Guidelines: Otorhinolaryngology–Head and Neck Surgery Volume” (1); “Practical Otorhinolaryngology” (1); “Practical Otorhinolaryngology–Head and Neck Surgery” (1); “Practical Internal Medicine” (1); “Modern Diagnosis and Treatment in Pediatric Traditional Chinese Medicine” (1); “Introduction to the Four-Grade Weighted Scoring Method for Clinical Diagnosis and Evaluation of Therapeutic Effect” (1)	5
Dry and burning throat	37	10	“Guiding Principles for Clinical Research of New Chinese Medicines” (27); “Otorhinolaryngology in Traditional Chinese Medicine” (10); “Criteria for Diagnosis and Therapeutic Effect of Diseases and Syndromes in Traditional Chinese Medicine” (3); “Otorhinolaryngology” (1); “Diagnostic Otorhinolaryngology” (1); “Clinical Practice Guidelines: Otorhinolaryngology–Head and Neck Surgery Volume” (1); “Practical Otorhinolaryngology” (1); “Practical Internal Medicine” (1); “Introduction to the Four-Grade Weighted Scoring Method for Clinical Diagnosis and Evaluation of Therapeutic Effect” (1); “Guidelines for Diagnosis and Treatment of Otorhinolaryngology–Head and Neck Surgery Diseases” (1)	1
Cough	25	8	“Guiding Principles for Clinical Research of New Chinese Medicines” (19); “Criteria for Diagnosis and Therapeutic Effect of Diseases and Syndromes in Traditional Chinese Medicine” (5); “Practical Internal Medicine” (2); “Otorhinolaryngology in Traditional Chinese Medicine” (2); “Introduction to the Four-Grade Weighted Scoring Method for Clinical Diagnosis and Evaluation of Therapeutic Effect” (1); “Pediatrics of Traditional Chinese Medicine” (1); “Criteria for Diagnosis and Therapeutic Effect of Otorhinolaryngologic Diseases and Syndromes in Traditional Chinese Medicine” (1); “Zhu Futang’s Practical Pediatrics” (1)	2
Tumor necrosis factor-alpha (TNF-α)	21	1	Enzyme-linked immunosorbent assay (ELISA) (20)	1
Interleukin-6 (IL-6)	18	1	Enzyme-linked immunosorbent assay (ELISA) (18)	0
Dry throat	17	5	“Guiding Principles for Clinical Research of New Chinese Medicines” (8); “Criteria for Diagnosis and Therapeutic Effect of Diseases and Syndromes in Traditional Chinese Medicine” (2); “Otorhinolaryngology in Traditional Chinese Medicine” (2); “Clinical Practice Guidelines: Otorhinolaryngology–Head and Neck Surgery Volume” (1); “Criteria for Diagnosis and Therapeutic Effect of Otorhinolaryngologic Diseases and Syndromes in Traditional Chinese Medicine” (1)	3
Pharyngeal redness and swelling	16	8	“Guiding Principles for Clinical Research of New Chinese Medicines” (7); “Criteria for Diagnosis and Therapeutic Effect of Diseases and Syndromes in Traditional Chinese Medicine” (2); “Zhu Futang’s Practical Pediatrics” (2); “Practical Otorhinolaryngology” (1); “Modern Diagnosis and Treatment in Pediatric Traditional Chinese Medicine” (1); “Pediatrics of Traditional Chinese Medicine” (1); “Otorhinolaryngology in Traditional Chinese Medicine” (1); “Guidelines for Diagnosis and Treatment of Otorhinolaryngology–Head and Neck Surgery Diseases” (1)	2
Thirst	15	6	“Guiding Principles for Clinical Research of New Chinese Medicines” (10); “Criteria for Diagnosis and Therapeutic Effect of Diseases and Syndromes in Traditional Chinese Medicine” (3); “Otorhinolaryngology in Traditional Chinese Medicine” (2); “Pediatrics of Traditional Chinese Medicine” (1); “Guidelines for Diagnosis and Treatment of Otorhinolaryngology–Head and Neck Surgery Diseases” (1); “Practical Internal Medicine” (1)	2
TCM syndrome efficacy	15	6	“Guiding Principles for Clinical Research of New Chinese Medicines” (13); “Criteria for Diagnosis and Therapeutic Effect of Diseases and Syndromes in Traditional Chinese Medicine” (2); “Clinical Integrative Chinese and Western Otorhinolaryngology” (1); “Otorhinolaryngology” (1); “Otorhinolaryngology in Traditional Chinese Medicine” (1); “Pediatrics of Traditional Chinese Medicine” (1)	1
TCM syndrome score	13	3	“Guiding Principles for Clinical Research of New Chinese Medicines” (12); “Otorhinolaryngology in Traditional Chinese Medicine” (1); “Criteria for Diagnosis and Therapeutic Effect of Diseases and Syndromes in Traditional Chinese Medicine” (1)	0
Visual analogue scale (VAS)	11	1	Visual analogue scale (VAS) (11)	0
White blood cell count (WBC)	11	1	Automated biochemical analyzer (1)	10
Adverse events	11	3	Common Terminology Criteria for Adverse Events (CTCAE), version 5.0 (1); “Modern Diagnosis and Treatment in Pediatric Traditional Chinese Medicine” (1); “Practical Internal Medicine” (1)	8
Foreign body sensation in the throat	11	5	“Guiding Principles for Clinical Research of New Chinese Medicines” (5); “Criteria for Diagnosis and Therapeutic Effect of Diseases and Syndromes in Traditional Chinese Medicine” (3); “Practical Otorhinolaryngology–Head and Neck Surgery” (1); “Otorhinolaryngology in Traditional Chinese Medicine” (1); “Guidelines for Diagnosis and Treatment of Otorhinolaryngology–Head and Neck Surgery Diseases” (1)	2
Time to recovery	11	4	“Guiding Principles for Clinical Research of New Chinese Medicines” (5); “Criteria for Diagnosis and Therapeutic Effect of Diseases and Syndromes in Traditional Chinese Medicine” (3); “Diagnostic Otorhinolaryngology” (1); “Diagnostic Otorhinolaryngology” (1)	4
Dry stool	11	3	“Guiding Principles for Clinical Research of New Chinese Medicines” (10); “Criteria for Diagnosis and Therapeutic Effect of Diseases and Syndromes in Traditional Chinese Medicine” (2); “Pediatrics of Traditional Chinese Medicine” (1)	0

**TABLE 3:** Search strategy for PubMed.

### Traditional Chinese medicine
#1 “traditional Chinese medicine” [MeSH]
#2 “Chinese patent medicine” [MeSH]
#3 “traditional Chinese medicine injection” [MeSH]
#4 (“traditional Chinese medicine*” OR “Chinese patent medicine*” OR “traditional Chinese medicine injection*” OR “TCM”) [TIAB]
#5 (“particle*” OR “granule*” OR “Keli” OR “capsule*” OR “Jiaonang” OR “oral liquid*” OR “oral liquids*” OR “oral solution*” OR “san” OR “syrup*” OR “sirop*” OR “Pill*” OR “injection*” OR “injectable*” OR “injections*” OR “injectables*” OR “Pian*” OR “wan” OR “heji” OR “mixture” OR “dropping pill” OR “drop pill” OR “tangjiang” OR “dan” OR “aerosol” OR “spray” OR “qiwuji” OR “decoction” OR “Yin”) [TIAB]
#6= “#1” OR “#2” OR “#3” OR “#4” OR “#5”
### Study types
#7 “Randomized Controlled Trial” [Publication Type]
#8 “Randomized Controlled Trials as Topic” [MeSH]
#9 “Clinical Trial” [Publication Type]
#10 (“randomized controlled study” OR “randomized controlled trial” OR “randomized study” OR “randomized trial” OR “randomized placebo-controlled study” OR “randomized placebo-controlled trial” OR “randomized placebo controlled” OR “randomized placebo-controlled” OR “randomized double-blin*” OR “randomized double blin*”) [TIAB]
#11= “#7” OR “#8” OR “#19” OR “#10”
### acute pharyngitis
#12 “acute pharyngitis” [MeSH]
#13 (“acute pharyngitis” OR “Sore Throat*”)[TIAB]
#14= “#12” OR “#13”
#15= “#6” AND “#11” AND “#14”

**TABLE 4:** Mapping of original outcome terms to standardized outcome labels and outcome domains.

Original outcome term	Standardized outcome label	Outcome domains
Outcome dictionary		
efficacy	Clinical efficacy	Symptoms/Signs
efficiency		
therapeutic effect		
Clinical efficacy		
Sore throat	Sore throat	
Throat pain		
pharyngalgia		
throatache		
Fever	Fever	
Pharyngeal congestion	Pharyngeal congestion	
Throat congestion		
Congestion of pharyngeal mucosa		
Dry and burning throat	Dry and burning throat	
Dry throat burning		
Cough	Cough	
tussis		
begma		
Pharyngeal redness and swelling	Pharyngeal redness and swelling	
Dry throat	Dry throat	
Thirst	Thirst	
dry mouth		
VAS	VAS	
Foreign body sensation in the throat	Foreign body sensation in the throat	
Dry stool	Dry stool	
Constipation		
Difficulty swallowing	Difficulty swallowing	
Swallowing discomfort		
Yellow urine	Yellow urine	​
dark urine		
Coughing and expectoration	Coughing and expectoration	
Abnormal tongue image	Abnormal tongue image	
Headache	Headache	
Cephalalgia		
throat itch	Itchy throat	
Itchy throat		
hoarse voice	Hoarseness	
Hoarseness		
Symptoms	Symptoms	​
Enlargement of submandibular lymph nodes	Enlargement of submandibular lymph nodes	
Abnormal pulse condition	Abnormal pulse condition	
Uvula edema	Uvula edema	
Redness and swelling of the lateral pharyngeal cord	Redness and swelling of the lateral pharyngeal cord	​
Redness and swelling of follicles on the posterior pharyngeal wall	Redness and swelling of follicles on the posterior pharyngeal wall	
Main symptoms	Main symptoms	
Secondary symptoms	Secondary symptoms	
Aversion to cold	Aversion to cold	
Expectoration	Expectoration	
Pharyngeal examination	Pharyngeal examination	
Astriction	Astriction	
Decreased secretion	Decreased secretion	
Tenderness of submandibular lymph nodes	Tenderness of submandibular lymph nodes	
Halitosis	Halitosis	
Oral herpes	Oral herpes	
Lymph node enlargement	Lymph node enlargement	
Edema of soft palate and velar	Edema of soft palate and velar	
Thick phlegm	Thick phlegm	
Swelling and swelling of the uvula	Swelling and swelling of the uvula	
Pharyngeal signs	Pharyngeal signs	
Redness and swelling of pharyngeal mucosa/uvula	Redness and swelling of pharyngeal mucosa/uvula	​
Symptoms and signs	Symptoms and signs	
EAT10 rating	EAT10 rating	
Enlarged tonsils	Enlarged tonsils	
Swelling and pain of submandibular lymph nodes	Swelling and pain of submandibular lymph nodes	
Results of laryngoscopy examination	Results of laryngoscopy examination	
Mental restlessness or fatigue	Mental restlessness or fatigue	
Degree of damage to oropharyngeal mucosa	Degree of damage to oropharyngeal mucosa	
Abnormal pulse and fingerprint	Abnormal pulse and fingerprint	
Hot eyes	Hot eyes	
Pus plug	Pus plug	
Systemic symptoms	Systemic symptoms	
Loss of appetite	Loss of appetite	​
Headache and body aches	Headache and body aches	
Discomfort in the throat	Discomfort in the throat	
Pharyngeal lymphoid follicles	Pharyngeal lymphoid follicles	
Dry throat and sore throat	Dry throat and sore throat	
time to recovery	time to recovery	
TNF-α	TNF-α	Physical and chemical examination
IL-6	IL-6	
WBC	WBC	
CRP	CRP	
IL-1β	IL-1β	
CD4^+^	CD4^+^	
hs-CRP	hs-CRP	
CD3^+^	CD3^+^	
CD8^+^	CD8^+^	
SlgA	SlgA	
CD4+/CD8+	CD4+/CD8+	
IL-2	IL-2	
Pharynx swab culture	Pharynx swab culture	​
Neutrophil	Neutrophil	
IL-8	IL-8	
Th1	Th1	
Th1/Th2	Th1/Th2	
INF-γ	INF-γ	
ALT	ALT	
RBC	RBC	
GR	GR	
LY	LY	
ECG	ECG	
VCAM-1	VCAM-1	
Hb	Hb	
Cr	Cr	
BUN	BUN	
IL-1	IL-1	
IL-4	IL-4	
IL-5	IL-5	
Th2	Th2	
γ-GT	γ-GT	
AST	AST	​
MMP-9	MMP-9	
ALP	ALP	
PCT	PCT	
FPG	FPG	
LEU	LEU	
Urine Routine Examination	Urine Routine Examination	
PRO	PRO	
GLU	GLU	
BLD	BLD	
Lysozyme	Lysozyme	
Abnormal laboratory examination	Abnormal laboratory examination	
EOS	EOS	
SRT	SRT	
PLT	PLT	
TBIL	TBIL	
TCM Symptoms and Efficacy	TCM Syndrome Efficacy	TCM Syndrome
TCM Syndrome Efficacy		
TCM Efficacy		
TCM Syndrome Score	TCM Syndrome Score	
TCM Rating		
TCM Symptom Score		
Wind-heat syndrome	Wind-heat syndrome	
Lung stomach heat syndrome	Lung stomach heat syndrome	
Spleen and stomach damp heat syndrome	Spleen and stomach damp heat syndrome	
BPI rating	BPI rating	Quality of life
mood	mood	
daily work	daily work	
daily life	daily life	
Life interests	Life interests	
slumber	slumber	
Walking ability	Walking ability	
Relationship with others	Relationship with others	
Cost-effectiveness ratio (C-E)	Cost-effectiveness ratio (C-E)	Economic evaluation
indirect cost	indirect cost	
Onset time	Onset time	
Cost of experimental drugs	Cost of experimental drugs	
Medical cost	Medical cost	
Incremental cost-effectiveness ratio	Incremental cost-effectiveness ratio	
Direct non-medical costs	Direct non-medical costs	
Total direct medical costs	Total direct medical costs	
total cost	total cost	
Adverse event	Adverse event	security event

**FIGURE 8:**
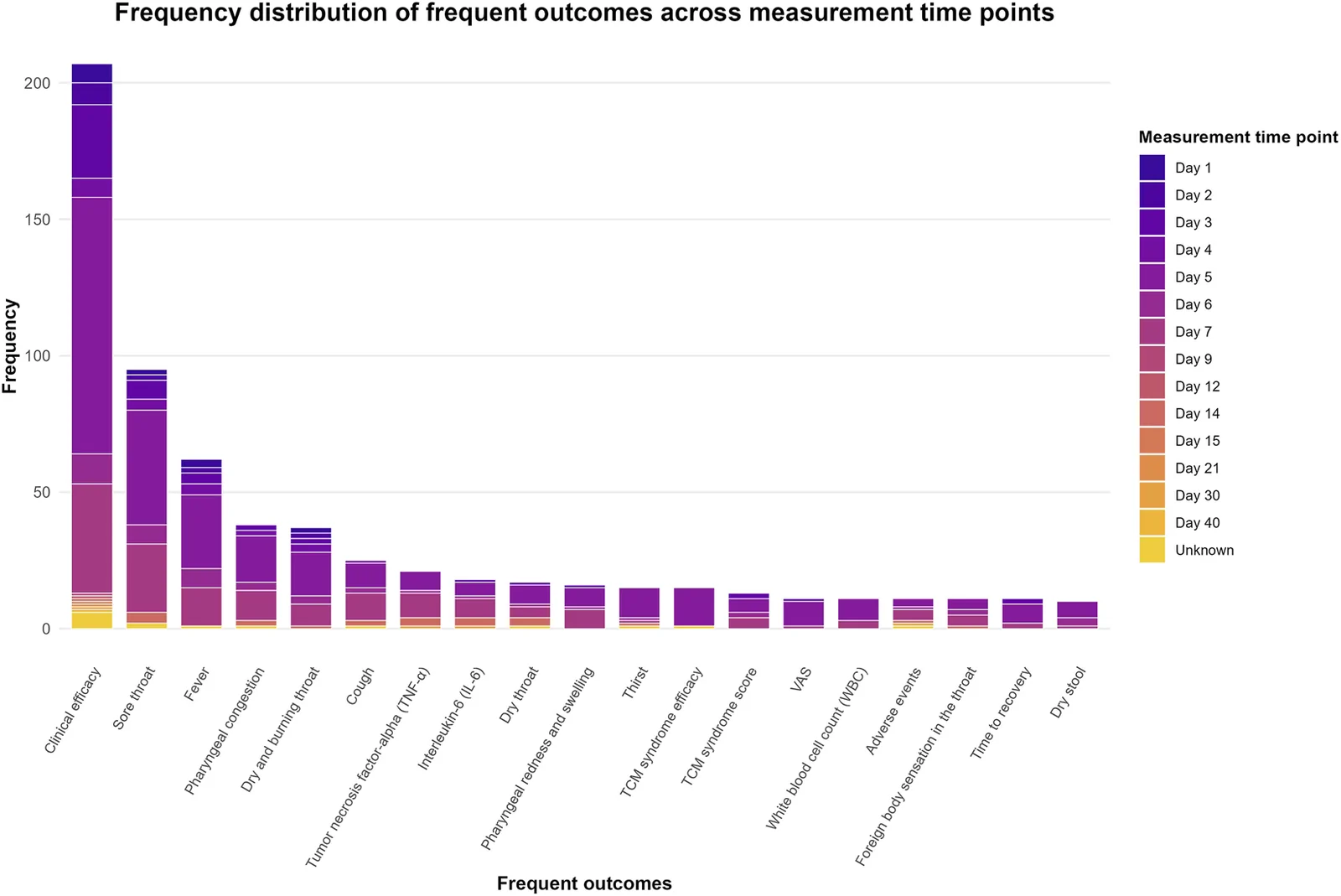
Stacked bar chart illustrating the distribution of measurement time points for outcomes with a reporting frequency greater than 10.

Considerable variability was also identified in the timing of outcome assessment among outcomes with a reporting frequency greater than 10 (Figure 8). Although most evaluations were conducted at the end of the treatment course, the specific time points varied markedly across studies, ranging from early assessments (e.g., day 1–3) to later follow-up points extending beyond 7 days or even longer durations. In addition, some trials adopted multiple assessment time points within the same study, further increasing heterogeneity. Notably, a subset of studies did not clearly specify the timing of outcome measurement (reported as “NK”), which limits interpretability and comparability. Overall, the lack of standardized assessment time points introduces an additional layer of inconsistency, potentially undermining the synthesis of evidence and highlighting the need for harmonized timing frameworks in future research.

## Discussion

The findings of this review indicate substantial heterogeneity in outcome selection and reporting across randomized controlled trials of traditional Chinese medicine (TCM) for acute pharyngitis, largely driven by the limited overlap in outcomes measured between studies. Although six overarching outcome domains and a large number of individual outcomes were identified, no single study captured all domains, and most trials reported outcomes within only one or two domains. This pattern suggests not merely incomplete coverage, but a lack of consistency in outcome prioritization across the evidence base. Symptom- and sign-based outcomes were universally reported and clearly dominated the evaluation framework, whereas other domains—including physical and chemical examinations, TCM syndrome measures, safety outcomes, economic evaluation, and particularly quality of life—were reported far less frequently. In addition, even within commonly assessed domains, only a small subset of outcomes (e.g., clinical efficacy and key symptom indicators) were repeatedly measured across studies, while the majority of outcomes were reported sporadically and with low frequency. Taken together, this imbalance between highly concentrated core outcomes and a long tail of infrequently reported measures highlights a fragmented outcome landscape in this field. Such variability limits comparability across studies and complicates evidence synthesis, underscoring the need for greater standardization in outcome definition, selection, and reporting in future TCM trials for acute pharyngitis.

The predominance of symptom- and sign-based outcomes was expected given the clinical nature of acute pharyngitis, where short-term relief of throat discomfort and visible inflammatory signs represents the primary therapeutic goal. Frequently reported outcomes such as sore throat, fever, and pharyngeal congestion are closely aligned with disease presentation and are readily assessable in routine practice. The widespread use of composite indicators such as overall clinical efficacy further reflects a preference for global measures that integrate multiple symptom dimensions into a single endpoint, an approach commonly adopted in clinical trials to capture overall treatment effects across related outcomes (Walker et al., 2024). However, this strong emphasis on clinician-assessed symptoms also suggests a continued reliance on a predominantly biomedical evaluation framework, with comparatively limited incorporation of patient-reported outcomes, which are considered the gold standard for capturing symptoms, quality of life, and overall wellbeing from the patient perspective (Puccini et al., 2025).

The increasing use of physical and chemical examination outcomes, particularly in more recent studies, may indicate a shift toward incorporating ostensibly objective biomarkers. Nevertheless, in the context of a largely self-limiting condition such as acute pharyngitis, the clinical relevance of routinely measuring inflammatory markers (e.g., CRP, leukocyte counts) warrants careful consideration. It remains unclear whether the inclusion of such indicators meaningfully enhances the assessment of treatment effectiveness or instead reflects a tendency to prioritize measurable laboratory endpoints over patient-important outcomes, potentially introducing unnecessary complexity and cost. By contrast, TCM syndrome-related outcomes, although more frequently reported in earlier studies, appear less prominent in recent research. This trend may not solely reflect changing methodological preferences, but also the inherent challenges associated with standardizing syndrome differentiation. The lack of universally accepted and validated measurement instruments for TCM syndrome assessment limits comparability across studies and reduces their utility in evidence synthesis, as inconsistency in diagnostic criteria and evaluation methods has been widely recognized as a major barrier to standardization and evidence-based research in this field (Zhenhan et al., 2024).

From a methodological perspective, the approach employed by Yang et al. (2026) is highly instructive. In their retrospective study of sepsis, clinical characteristics, traditional Chinese medicine (TCM) syndrome patterns, and laboratory biomarkers were systematically integrated to evaluate disease dynamics and patient responses. Although sepsis differs substantially from pharyngitis in clinical severity and endpoints, their framework highlights the value of harmonizing subjective clinical presentations with objective pathophysiological markers. Compared with this multidimensional approach, the pharyngitis trials included in our review exhibited a more fragmented outcome structure, with insufficient crosstalk between macro-level symptoms, traditional TCM syndrome characteristics, and micro-level laboratory evaluations. Therefore, future Core Outcome Set (COS) development for TCM treatment of pharyngitis should retain clinically important symptom-related outcomes while also incorporating objective inflammatory or immune indicators, safety outcomes, and health-related quality of life within a more balanced and standardized framework.

In addition, the near absence of quality-of-life measures and the limited reporting of economic outcomes highlight important gaps in the current evidence base. Despite the typically short disease course, acute pharyngitis can substantially affect swallowing, sleep, and daily activities, underscoring the importance of incorporating patient-centered outcomes. Similarly, given the widespread use of healthcare resources and concerns regarding inappropriate antibiotic prescribing, the lack of economic evaluation restricts understanding of the broader value of TCM interventions. Taken together, these patterns suggest that outcome selection in this field remains largely focused on immediate clinical manifestations, with insufficient attention to patient-centered and health system–level considerations.

Although used in the included studies, the Brief Pain Inventory (BPI) may not be the optimal tool for assessing quality-of-life outcomes in acute pharyngitis. This reflects a broader mismatch between generic pain-interference instruments and the disease-specific burden of acute pharyngitis. The BPI can capture the impact of pain on daily functioning and psychosocial wellbeing, but acute pharyngitis is characterized by a short and rapidly changing clinical course, involving localized symptoms, such as swallowing pain, throat irritation, and voice changes, as well as their immediate functional disruptions to eating, sleeping, and daily activities. These dimensions are only partially represented by BPI interference items. Therefore, reliance on BPI-derived outcomes may underestimate or incompletely characterize patient-perceived benefit in acute pharyngitis, particularly when symptom resolution, functional recovery, and short-term treatment response are central to clinical decision-making. Although several disease-specific patient-reported instruments have been developed for related conditions, such as tonsil and adenoid disease, recurrent acute tonsillitis, or tonsillectomy outcomes, validated PROMs specifically designed for acute pharyngitis appear to remain limited. This highlights an important measurement gap in the field. Future studies should not only include patient-reported outcomes, but also evaluate whether the selected instruments demonstrate adequate content validity for the symptom profile and time course of acute pharyngitis. In the context of core outcome set development, this finding supports the need to develop, validate, or adapt disease-specific PROMs that can better capture the short-term, symptom-driven, and functionally disruptive nature of acute pharyngitis.

No outcomes were identified in the long-term prognosis domain. This may partly reflect the acute and often self-limiting nature of pharyngitis, as well as trial designs that tend to prioritize rapid symptom relief, immediate clinical response, and feasibility of follow-up. However, the absence of this domain is particularly important in the evaluation of TCM interventions. From a TCM perspective, treatment is not limited to relieving acute symptoms, but may also aim to restore balance, reduce susceptibility, prevent recurrence, and avoid progression from an acute to a chronic condition. Without follow-up beyond the immediate treatment period, it is difficult to determine whether TCM interventions offer sustained benefits or advantages in reducing recurrence and downstream healthcare use. This evidence gap is also relevant to broader clinical and public health concerns, such as repeated consultations and potential antibiotic overuse in acute pharyngitis. Future studies should therefore consider incorporating outcomes such as recurrence, sustained symptom resolution, subsequent healthcare utilization, antibiotic use, long-term safety, and patient-reported recovery experience, so as to better capture the potential long-term and patient-centered value of TCM interventions.

Among individual outcomes, overall clinical efficacy was the most frequently reported endpoint, being used in the vast majority of included studies. While this high prevalence underscores its central role in evaluating treatment effects, it also raises important methodological concerns. As a composite indicator, clinical efficacy typically aggregates multiple symptom changes into a single categorical judgment (Cordoba et al., 2010), which may obscure variation across individual components and limit interpretability (Ferreira-González et al., 2007). Nevertheless, its continued widespread use is likely attributable to its practicality and feasibility (Freemantle et al., 2003), as it provides a convenient summary measure that is relatively simple to implement in clinical settings without requiring complex or resource-intensive assessments. In comparison, previous reviews in other clinical fields have similarly reported a high reliance on composite or global endpoints, often driven by the need to capture overall treatment effects while maintaining feasibility in trial design. However, such approaches have also been critiqued for potentially masking clinically meaningful differences across individual outcomes and for lacking standardization in definition and measurement (Cordoba et al., 2010; Ferreira-González et al., 2007; Freemantle et al., 2003). From a conceptual perspective, the prominence of global clinical efficacy measures may also reflect an implicit alignment with holistic evaluation frameworks (Hu et al., 2025), particularly within TCM practice, where treatment effects are often interpreted in terms of overall symptom improvement rather than isolated endpoints (H et al., 2015). At the same time, the limited adoption of standardized patient-reported measures, such as visual analogue scales, suggests that this holistic orientation has not yet been translated into rigorously validated and patient-centered measurement approaches (Hu et al., 2025). Together, these findings highlight a tension between pragmatic outcome selection, traditional evaluation paradigms, and evolving expectations for methodological rigor in clinical research.

The predominance of symptom-based outcomes is both clinically justified and methodologically problematic. On the one hand, this pattern reflects a patient-centered orientation, capturing the perceived disease burden of pharyngitis (e.g., sore throat, fever, pharyngeal discomfort, dryness, and cough). Furthermore, the frequent reporting of TCM syndrome scores aligns with TCM clinical practice, where syndrome differentiation is central to therapeutic evaluation. On the other hand, over-reliance on symptom-based outcomes weakens the comparability of trial findings, especially when assessed using unstandardized or self-developed criteria. In our review, standardized tools (such as VAS) were reported far less frequently than general symptom descriptions. Moreover, objective inflammatory markers (e.g., TNF-α, IL-6, WBC, CRP) and vital safety outcomes were substantially underreported, indicating a lack of risk-benefit and mechanistic evaluation. These findings suggest that future Core Outcome Set (COS) development should retain patient-centered and TCM-specific outcomes, while mandating the inclusion of standardized symptom scales, objective biomarkers, and safety metrics.

The focus on sore throat, fever, and pharyngeal congestion is expected, as these are the primary reasons patients seek medical care for pharyngitis. Consequently, current trial designs have prioritized the relief of acute, visible symptoms over a comprehensive assessment of overall disease burden. While clinically relevant, the heavy reliance on broad “clinical efficacy” categories—often based on subjective physician judgment—limits comparability across studies. Notably, the rare use of standardized patient-reported outcome measures (PROMs), such as the Visual Analogue Scale (VAS), indicates a failure to accurately capture the patients’ subjective experience. Furthermore, the low reporting frequency of symptoms like dysphagia and hoarseness suggests that functional impairments impacting quality of life remain marginalized. This likely reflects the difficulty in standardizing these metrics, driving researchers toward more conventional inflammatory symptoms. Ultimately, these findings highlight a critical methodological gap. Future Core Outcome Set (COS) development must retain core acute symptoms but urgently incorporate validated PROMs and functional endpoints to comprehensively evaluate therapeutic efficacy.

Economic evaluation outcomes were also rarely reported, despite the high prevalence of acute pharyngitis and the associated use of healthcare resources. Given the widespread use of therapeutic interventions—including ongoing concerns regarding unnecessary antibiotic prescribing—the absence of cost-related outcomes limits understanding of the broader value and potential health system implications of these treatments. This gap suggests that current research remains largely focused on short-term clinical effects, with insufficient integration of health economic considerations that are increasingly important for informing policy and resource allocation. At a broader level, outcomes related to long-term prognosis, patient-reported wellbeing, and system-level or environmental factors were largely absent. This may partly reflect the typically short disease course of acute pharyngitis, as well as trial designs that prioritize feasibility and immediate symptom relief. However, there is growing recognition that intervention evaluation should extend beyond short-term clinical endpoints to consider wider health, behavioral, and system-level impacts, including healthcare utilization, patient experience, and downstream consequences. Overall, these findings suggest that future studies should move beyond narrowly defined clinical outcomes to incorporate patient-centered, safety, economic, and system-level measures. Importantly, outcome selection should also reflect the perspectives of multiple stakeholders. The current emphasis on researcher-driven endpoints may not fully capture outcomes that matter most to patients, caregivers, and healthcare providers, highlighting the need for more inclusive and consensus-based approaches to outcome development.

Since only a small proportion of included trials reported safety events, relying solely on published data risks yielding an incomplete candidate list for subsequent COS development. This limitation is particularly critical for TCM interventions, where safety profiles encompass not only general adverse events but also preparation-specific reactions, herb–drug interactions, and organ-function abnormalities. To address this, we will supplement our candidate list by consulting additional sources, including package inserts of the included TCM preparations, pharmacovigilance data, national or regional essential-medicines catalogues, relevant clinical guidelines, and expert/stakeholder suggestions. This strategy is expected to minimize under-ascertainment and ensure the Delphi survey includes clinically relevant and patient-important safety domains applicable to TCM for pharyngitis.

Correlation analysis revealed distinct, clinically interpretable outcome clusters. Jointly mediating inflammatory signaling, IL-6, TNF-α, and IL-1β clustered strongly as a coherent biomarker panel, reflecting their frequent co-evaluation in mechanistic TCM research. For instance, despite pathophysiological differences from pharyngitis, studies on herbal interventions in critical care (Yang et al., 2026) demonstrate the value of evaluating these related cytokines as an integrated cluster rather than isolated variables. Similarly, T-cell subsets (CD3^+^,CD4^+^, CD8^+^, and the CD4^+^/CD8^+^ ratio) closely clustered as a standardized immunological panel, consistent with the growing trend of pairing biological markers with clinical scales (Jin et al., 2026). Regarding clinical presentations, moderate clustering also emerged among symptoms like sore throat, fever, and pharyngeal congestion. Ultimately, these empirical patterns suggest that future core outcome set (COS) development for TCM treatment of pharyngitis must move away from disconnected domains. Instead, a balanced framework should integrate patient-perceived symptoms, TCM-specific responses, objective biological markers, and safety outcomes to improve trial comparability and risk-benefit interpretability.

The characterization of TCM interventions was often insufficient in the original studies. Although most trials reported the name and dosage of the Chinese patent medicine, decoction, or hospital preparation, key information needed to define the preparation itself was often incomplete. This is particularly problematic for multi-component herbal preparations, because differences in composition, source materials, processing, extraction, and manufacturing standards may alter their chemical profiles and, consequently, their pharmacological and clinical effects. As a result, preparations with the same or similar names cannot necessarily be assumed to be equivalent across studies, and the comparability of interventions should be interpreted with caution. Future clinical studies of TCM and other herbal medicinal products should follow ConPhyMP (Heinrich et al., 2022) or similar reporting guidelines and provide standardized information on taxonomically validated species names, plant families, medicinal parts, formulation composition, manufacturer, batch number, preparation and extraction procedures, quality-control methods, and chemical standardization markers where applicable. More transparent reporting would improve the reproducibility, comparability, and interpretability of clinical evidence on herbal and multi-component medicinal preparations.

The substantial heterogeneity observed in outcome selection and reporting poses a significant challenge for the evaluation and synthesis of evidence in this field. The wide variation in both outcome domains and individual measures, together with the limited overlap across studies, restricts comparability and undermines the reliability of cross-trial interpretation. This inconsistency not only complicates meta-analysis and evidence integration, but may also contribute to research inefficiency and waste, as findings cannot be readily combined or meaningfully compared. These findings underscore the need for greater standardization in outcome selection, definition, and measurement in trials of traditional Chinese medicine for acute pharyngitis. In this context, the development of a core outcome set (COS) represents a pragmatic and internationally recognized approach to address current limitations by establishing a minimum set of outcomes that should be consistently measured and reported across studies. Importantly, beyond defining what to measure, future work should also consider how outcomes are measured, including the selection of valid, reliable, and responsive measurement instruments in line with established methodological frameworks such as COSMIN, to further reduce heterogeneity at the measurement level.

Discrepancies between registered and published outcomes may undermine the reliability of the evidence base by introducing selective outcome reporting. In this review, all prespecified outcomes in the eligible registration records were reported in the corresponding articles, but several publications also included additional non-prespecified outcomes. If such outcomes are introduced or emphasized after trial conduct, they may overstate treatment effects, inflate the apparent diversity of reported outcomes, and complicate the interpretation of intervention benefits. Our comparison of trial registrations with published reports, strengthened the methodological rigor of this review by making these reporting patterns more transparent. Future trials should prospectively register clearly defined primary and secondary outcomes and explicitly justify any *post hoc* additions or deviations from the registered protocol.

This review represents the first stage in the development of such a COS. The outcomes identified herein provide a comprehensive foundation for subsequent consensus-building processes, including Delphi surveys and structured consensus meetings, conducted in accordance with methodological guidance such as that recommended by the COMET Initiative. Crucially, these processes should involve a broad range of stakeholders, including clinicians, researchers, patients, and policymakers. Such inclusive engagement is essential to ensure that the resulting COS reflects not only clinical priorities but also patient-valued outcomes, such as quality of life, as well as health system considerations, including economic impact.

The establishment of a standardized and stakeholder-informed COS may also facilitate greater alignment of TCM clinical research with international reporting standards, thereby enhancing its transparency, comparability, and global relevance. In addition, the consistent use of predefined core outcomes has the potential to reduce selective outcome reporting and improve the completeness of evidence, ultimately strengthening the credibility and usability of research findings. Collectively, these efforts will support more robust evidence synthesis and contribute to the advancement of high-quality, patient-centered, and policy-relevant research in this field.

The present review has several notable strengths. First, by restricting inclusion to randomized controlled trials (RCTs), this study prioritizes evidence derived from rigorous experimental designs, thereby enhancing the internal validity of the findings and reducing the influence of confounding factors commonly encountered in observational research. Second, a comprehensive and systematic search strategy was implemented across multiple databases, with predefined eligibility criteria and standardized procedures for study selection and data extraction, which helps to minimize selection bias and improve reproducibility. Third, this review places particular emphasis on clinically relevant outcomes in patients with acute pharyngitis, allowing for a more meaningful interpretation of the therapeutic value of traditional Chinese medicine interventions in real-world practice. In addition, the structured synthesis of outcome measures across studies contributes to a clearer understanding of commonly assessed endpoints and highlights areas of consistency and divergence in the existing evidence base. Collectively, these methodological strengths enhance the reliability and interpretability of the current findings, and provide a valuable foundation for future research in this field.

### Limitations

Our review has several limitations that inform the interpretation of these findings and the subsequent development of a core outcome set. First, outcome identification relied mainly on published reports and specific trial registries; therefore, outcomes that were incompletely reported, omitted, or published in non-target languages may not have been fully captured. Second, harmonizing diverse outcome terminology and assigning them to standardized domains inherently required reviewer judgment, which introduces potential subjective bias despite our consensus checks. Third, because we focused strictly on identifying and mapping reported outcomes, we did not evaluate the measurement properties (e.g., validity, reliability) of the corresponding instruments; thus, a high reporting frequency does not necessarily equate to clinical importance. Finally, the inadequate reporting of TCM intervention details—such as variations in formulation, dosage, or syndrome differentiation—limited our ability to ensure strict comparability across similarly named treatments.

## Conclusions

This review demonstrates considerable inconsistency in the selection and reporting of outcomes across studies evaluating traditional Chinese medicine interventions for acute pharyngitis. Although a wide range of outcome domains and individual measures were identified, most were reported infrequently, indicating limited alignment in outcome prioritization across trials. Such variability constrains the comparability of findings and reduces the efficiency of evidence synthesis.

Given the high burden of acute pharyngitis and the extensive use of therapeutic interventions, improving the consistency and relevance of outcome reporting is essential. The findings of this review provide a foundation for the development of a core outcome set (COS) tailored to this field, with the aim of promoting more standardized, transparent, and patient-relevant outcome assessment. The implementation of a COS is expected to enhance the quality and interpretability of future research, support more robust evidence synthesis, and ultimately contribute to more informed clinical and policy decision-making.

## Data Availability

The original contributions presented in the study are included in the article/Supplementary Material, further inquiries can be directed to the corresponding authors.

## References

[B1] Alexander L. Cooper K. Peters M. D. J. Tricco A. C. Khalil H. Evans C. (2024). Large scoping reviews: managing volume and potential chaos in a pool of evidence sources. J. Clin. Epidemiol. 170, 111343. 10.1016/j.jclinepi.2024.111343 38582403

[B46] Arksey H. O’Malley L. (2005). Scoping studies: towards a methodological framework. Int. J. Soc. Res. Methodol. 8 (1), 19–32. 10.1080/1364557032000119616

[B2] Bossuyt P. M. Loh T. P. Bell K. Lord S. Horvath A. R. (2026). Frameworks of health outcomes for performance specifications in laboratory medicine. Clin. Chem. 72 (5), 546–553. 10.1093/clinchem/hvag014 41740616

[B3] Braga L. H. Farrokhyar F. Dönmez M. İ. Nelson C. P. Haid B. Herbst K. (2025). Randomized controlled trials - the what, when, how and why. J. Pediatr. Urol. 21 (2), 397–404. 10.1016/j.jpurol.2024.11.021 39701869

[B4] Catic T. Kapo B. Pintol Z. Skopljak A. Cengic A. Gojak R. (2018). An instrument for rating quality of life related to sore throat in patients suffering from acute pharyngitis or tonsillitis. Mater Sociomed. 30 (1), 43–48. 10.5455/msm.2018.30.43-48 30429687 PMC6234653

[B5] Chalmers I. Bracken M. B. Djulbegovic B. Garattini S. Grant J. Gülmezoglu A. M. (2014). How to increase value and reduce waste when research priorities are set. Lancet 383 (9912), 156–165. 10.1016/S0140-6736(13)62229-1 24411644

[B6] Cordoba G. Schwartz L. Woloshin S. Bae H. Gøtzsche P. C. (2010). Definition, reporting, and interpretation of composite outcomes in clinical trials: systematic review. BMJ 341, c3920. 10.1136/bmj.c3920 20719825 PMC2923692

[B7] Devane D. Briel M. Bhagani S. Boesten N. Buchholz S. De Luca E. C. (2025). Protocol for a core outcome set for pharmacological treatments in hospitalised patients with acute viral respiratory infections (COSAVRI). PLoS One 20 (9), e0330288. 10.1371/journal.pone.0330288 40924748 PMC12419614

[B8] Fan X. Yuan M. Li G. Kang T. Li P. Xie Q. (2025). Xujiang Xie's bloodletting therapy combined with Qingyan Lige decoction for acute pharyngitis with lung-stomach heat accumulation: a randomized controlled trial. Zhongguo Zhen Jiu 45 (11), 1565–1570. 10.13703/j.0255-2930.20241120-0001 41261540

[B9] Fang M. Kong L. Ji G. Pu F. Su Y. Li Y. (2024). Chinese medicine Phragmites communis (Lu Gen) for acute respiratory tract infections: a systematic review and meta-analysis of randomized controlled trials. Front. Pharmacol. 15, 1242525. 10.3389/fphar.2024.1242525 38510651 PMC10953292

[B10] Ferreira-González I. Busse J. W. Heels-Ansdell D. Montori V. M. Akl E. A. Bryant D. M. (2007). Problems with use of composite end points in cardiovascular trials: systematic review of randomised controlled trials. BMJ 334 (7597), 786. 10.1136/bmj.39136.682083.AE 17403713 PMC1852019

[B11] Freemantle N. Calvert M. Wood J. Eastaugh J. Griffin C. (2003). Composite outcomes in randomized trials: greater precision but with greater uncertainty? JAMA 289 (19), 2554–2559. 10.1001/jama.289.19.2554 12759327

[B12] Gorst S. L. Prinsen C. A. C. Salcher-Konrad M. Matvienko-Sikar K. Williamson P. R. Terwee C. B. (2020). Methods used in the selection of instruments for outcomes included in core outcome sets have improved since the publication of the COSMIN/COMET guideline. J. Clin. Epidemiol. 125, 64–75. 10.1016/j.jclinepi.2020.05.021 32470621

[B13] Hedin K. Thorning S. van Driel M. L. (2023). Different antibiotic treatments for group A streptococcal pharyngitis. Cochrane Database Syst. Rev. 11 (11), CD004406. 10.1002/14651858.CD004406.pub6 37965935 PMC10646936

[B47] Heinrich M. Jalil B. Abdel-Tawab M. Echeverria J. Kulić Ž Z. McGaw L. J. (2022). Best Practice in the chemical characterisation of extracts used in pharmacological and toxicological research—The ConPhyMP—Guidelines. Front. Pharmacol. 13, 953205. 10.3389/fphar.2022.953205 36176427 PMC9514875

[B14] Hu Y. Wang Z. Ni K. Yang J. (2025). Challenges in traditional Chinese medicine clinical trials: how to balance personalized treatment and standardized research? Ther. Clin. Risk Manag. 21, 1085–1094. 10.2147/TCRM.S523279 40666695 PMC12262083

[B15] Jianxin W. Rui S. Fengwen Z. (2021). Technical specifications for the development of core indicators for clinical trials of traditional Chinese medicine. Chin. J. Traditional Chin. Med. 36 (2), 924–928.

[B16] Jin N. Wang X. Lai M. Zou J. He L. Luo G. (2026). Elucidating the mechanism of fusu agent against sepsis-induced acute respiratory distress syndrome *via* an integrated approach combining network pharmacology, molecular dynamics simulations, and experimental validation. J. Inflamm. Res. 19, 572866. 10.2147/JIR.S572866 41913790 PMC13033274

[B17] Keyan L. Wei L. Haojia S. (2023). Traditional Chinese patent medicines and simple preparations treatment of acute pharyngitis data mining and mesh meta analysis. J. Otolaryngology Ophthalmol. Shandong Univ. 37 (4), 111–118.

[B18] Kirkham J. J. Dwan K. M. Altman D. G. Gamble C. Dodd S. Smyth R. (2010). The impact of outcome reporting bias in randomised controlled trials on a cohort of systematic reviews. BMJ 340, c365. 10.1136/bmj.c365 20156912

[B19] Kirkham J. J. Davis K. Altman D. G. Blazeby J. M. Clarke M. Tunis S. (2017). Core outcome Set-STAndards for development: the COS-STAD recommendations. PLoS Med. 14 (11), e1002447. 10.1371/journal.pmed.1002447 29145404 PMC5689835

[B20] Lin L. Xiao J. Wu L. Fan F. Feng W. Wei J. (2024a). Efficacy and safety of Kegan Liyan oral liquid patients with acute pharyngitis: a randomized, double-blinded, placebo-controlled, multi-center trial. Phytomedicine 134, 155960. 10.1016/j.phymd.2024.155960 39217655

[B21] Lin L. Xiao J. Wu L. Fan F. Feng W. Wei J. (2024b). Efficacy and safety of Kegan Liyan oral liquid for patients with acute pharyngitis: a randomized, double-blinded, placebo-controlled, multi-center trial. Phytomedicine 134, 155960. 10.1016/j.phymed.2024.155960 39217655

[B22] Liu K. Shi L. Song Y. Du Y. Yuan Y. Shi Z. (2025). Xiao'er Fengreqing oral liquid *versus* Xiao'er Yanbian granules for pediatric acute pharyngitis/tonsillitis (external wind heat syndrome): protocol and statistical analysis plan for a multi-center, randomized, double-blind, active drug-controlled trial. Front. Pharmacol. 16, 1625547. 10.3389/fphar.2025.1625547 40756995 PMC12314424

[B23] Luo H. Liao X. Wang Q. (2015). Application of Chinese medical syndrome scores in effectiveness evaluation: a critical appraisal of 240 randomized controlled trials. Chin. J. Integr. Traditional West. Med. 35 (10), 1261–1266. 26677681

[B24] Ma Y. Zhong C. Hu S. Cai Q. Guo S. (2021). Evaluation on immediate analgesic efficacy and safety of kai-hou-jian spray (children's type) in treating sore throat caused by acute pharyngitis and tonsillitis in children: study protocol for a randomized controlled trial. Trials 22 (1), 216. 10.1186/s13063-021-05148-1 33736674 PMC7977177

[B25] Malmberg S. Hess-Wargbaner M. Björk D. Jacobsson G. Ulleryd P. Gunnarsson R. (2026). Long-term effects of a multifaceted antimicrobial stewardship program, with audit and feedback, on acute sore throat in primary care - a randomized controlled trial. Int. J. Infect. Dis. 164, 108427. 10.1016/j.ijid.2026.108427 41581646

[B26] Mingsan M. Mengfan P. Baosong Z. (2017). Discussion on the advantages and problems of traditional Chinese medicine in treating acute pharyngitis. China J. Chin. Materia Medica 42 (19), 3819–3825. 10.19540/j.cnki.cjcmm.20170901.002 29235301

[B27] Mokkink L. B. de Vet H. C. W. Prinsen C. A. C. Patrick D. L. Alonso J. Bouter L. M. (2018). COSMIN risk of bias checklist for systematic reviews of patient-reported outcome measures. Qual. Life. Res. 27 (5), 1171–1179. 10.1007/s11136-017-1765-4 29260445 PMC5891552

[B28] Munn Z. Peters M. D. J. Stern C. Tufanaru C. Mcarthur A. Aromataris E. (2018). Systematic review or scoping review? Guidance for authors when choosing between a systematic or scoping review approach. BMC Med. Res. Methodol. 18 (1), 143. 10.1186/s12874-018-0611-x 30453902 PMC6245623

[B29] Pellegrino R. Timitilli E. Verga M. C. Guarino A. Iacono I. D. Scotese I. (2023a). Acute pharyngitis in children and adults: descriptive comparison of current recommendations from national and international guidelines future perspectives. Eur. J. Pediatr. 182 (12), 5259–5273. 10.1007/s00431-023-0211-w 37819417 PMC10746578

[B30] Pellegrino R. Timitilli E. Verga M. C. Guarino A. Iacono I. D. Scotese I. (2023b). Acute pharyngitis in children and adults: descriptive comparison of current recommendations from national and international guidelines and future perspectives. Eur. J. Pediatr. 182 (12), 5259–5273. 10.1007/s00431-023-05211-w 37819417 PMC10746578

[B31] Prinsen C. A. C. Vohra S. Rose M. R. Boers M. Tugwell P. Clarke M. (2016). How to select outcome measurement instruments for outcomes included in a Core Outcome Set - a practical guideline. Trials 17 (1), 449. 10.1186/s13063-016-1555-2 27618914 PMC5020549

[B32] Puccini A. Viscardi G. Ciani O. Efficace F. Piattelli A. Beretta G. D. (2025). Patient-reported outcomes (PROs) in clinical trials and in clinical practice: report from the XXI national conference of the Italian Association of Medical Oncology (AIOM). BMJ Oncol. 4 (1), e000783. 10.1136/bmjonc-2025-000783 40546927 PMC12182000

[B33] Rong W. Sumei S. Yi X. (2024). Expert consensus on integrated traditional Chinese and Western medicine diagnosis and treatment of pharyngitis and tonsillitis in children. Chin. J. Integr. Pediatr. 16 (5), 399–403.

[B34] Shi Y. Gu R. Liu C. Ni J. Wu T. (2007). Chinese medicinal herbs for sore throat. Cochrane Database Syst. Rev. (3), CD004877. 10.1002/14651858.CD004877.pub2 17636777

[B35] Tricco A. C. Lillie E. Zarin W. O'Brien K. K. Colquhoun H. Levac D. (2018). PRISMA extension for scoping reviews (PRISMA-ScR): checklist and explanation. Ann. Intern. Med. 169 (7), 467–473. 10.7326/M18-0850 30178033

[B36] Vincent M. T. Celestin N. Hussain A. N. (2004). Pharyngitis. Am. Fam. Physician 69 (6), 1465–1470. 15053411

[B37] Walker H. G. M. Brown A. J. Vaz I. P. Reed R. Schofield M. A. Shao J. (2024). Composite outcome measures in high-impact critical care randomised controlled trials: a systematic review. Crit. Care. 28 (1), 184. 10.1186/s13054-024-04967-3 38807143 PMC11134769

[B38] Williamson P. R. Altman D. G. Blazeby J. M. Clarke M. Devane D. Gargon E. (2012). Developing core outcome sets for clinical trials: issues to consider. Trials 13, 132. 10.1186/1745-6215-13-132 22867278 PMC3472231

[B39] Williamson P. R. Altman D. G. Bagley H. Barnes K. L. Blazeby J. M. Brookes S. T. (2017). The COMET handbook: version 1.0. Trials 18 (Suppl. 3), 280. 10.1186/s13063-017-1978-4 28681707 PMC5499094

[B40] Yang M. Chen T. Liu J. Wang X. Wang X. Xu X. (2026). Clinical characteristics and prognostic factors in patients with sepsis: a retrospective study. J. Cell. Mol. Med. 30 (2), e71005. 10.1111/jcmm.71005 41588929 PMC12835617

[B41] Yan-ning Z. Si-yuan R. Sheng-xuan Z. (2025). Expert consensus on traditional Chinese medicine diagnosis and efficacy evaluation of acute pharyngitis. Chin. J. Evid. Based Med. 25 (9), 993–1000.

[B42] Young A. E. Brookes S. T. Avery K. N. L. Davies A. Metcalfe C. Blazeby J. M. (2019). A systematic review of core outcome set development studies demonstrates difficulties in defining unique outcomes. J. Clin. Epidemiol. 115, 14–24. 10.1016/j.jclinepi.2019.06.016 31276780

[B43] Zhang S. Cui Y. Zhou X. Wang D. Yin J. Meng X. (2023). Efficacy of acupuncture on acute pharynx infections: a systematic review and meta-analysis. Med. Baltim. 102 (25), e34124. 10.1097/MD.0000000000034124 37352021 PMC10289600

[B44] Zhenhan W. Mingzhou R. Jieqiong F. (2024). Research progress on the evaluation methods of traditional Chinese medicine syndrome efficacy. China J. Chin. Materia Medica 49 (6), 1467–1473. 10.19540/j.cnki.cjcmm.20240103.501 38621930

[B45] Zonghai H. Jiaqing M. Ju L. Z F. Y X. J W. (2022). Practice and strategy of developing a traditional Chinese medicine syndrome efficacy evaluation Scale under the mode of disease syndrome integration. Chin. J. General Pract. 25 (20), 2513–2519.

