# Supplementary material for: Outcomes reported in randomized controlled trials of traditional Chinese medicine for acute pharyngitis: a scoping review
Source: Front Pharmacol. 2026 Aug 26;17:1872689. doi: 10.3389/fphar.2026.1872689 (PMC13553261; doi:10.3389/fphar.2026.1872689)
Supplement: Supplementary file 2 [file DataSheet1.docx]

| **Category** | **Intervention name(English/Chinese)** | **Intervention type** | **Number of studies** | **Dosage form** | **Administration route** | **Dosage & frequency** | **Treatment duration** | **Composition (herbs/ingredients)** | **Botanical Latin name (with authority)** | **Plant family** | **Plant part used** | **Pharmacopeial status(CP/ USP/none)** | **Manufacturer/source** | **Preparation/extraction method** | **Quality control /standardization markers** | **Batch number(if reported)** | **Taxonomic validation source (e.g.POWO/MPNS)** | **Reported in original trial(Y/N/partial)** |
| --- | --- | --- | --- | --- | --- | --- | --- | --- | --- | --- | --- | --- | --- | --- | --- | --- | --- | --- |
| Marketed Chinese patent medicines | Shufeng Jiedu Capsule / 疏风解毒胶囊 | Proprietary multi-herbal TCM formula (patent medicine) | 7 | Capsule | Oral | 4 capsules (0.52 g each), TID | 5–6 days | Tiger herb, Forsythia, Isatis root, Bupleurum, Patrinia, Verbena, Phragmites, Licorice | Reynoutria japonica Houtt.; Forsythia suspensa (Thunb.) Vahl; Isatis indigotica Fort.; Bupleurum chinense DC.; Patrinia scabiosaefolia Fisch. ex Trevir.; Verbena officinalis L.; Phragmites australis (Cav.) Trin. ex Steud.; Glycyrrhiza uralensis Fisch. ex DC. | Polygonaceae;Oleaceae;Brassicaceae;Apiaceae;Caprifoliaceae;Verbenaceae;Poaceae;Fabaceae | Rhizome/root; fruit; root; root; root; whole herb; rhizome; root | CP-listed proprietary formula (limited disclosure; not fully standardized in trials) | Anhui Jiren Pharmaceutical Co., Ltd. | NR (industrial proprietary formulation) | Not reported | NR | Not reported (no POWO/MPNS cited) | Partial |
| Marketed Chinese patent medicines | Ertong Qingyan Jiere Oral Liquid / 儿童清咽解热口服液 | Proprietary multi-herbal TCM formula (patent medicine) | 6 | Oral liquid | Oral | 1–3 y: 5 mL; 4–7 y: 10 mL; ≥7 y: 15 mL; TID | 5–7 days | Radix Bupleuri; Radix Scutellariae; Artificial Bovis Calculus; Herba Violae; Herba Patriniae; Herba Houttuyniae; Radix Phragmitis; Semen Phaseoli | Bupleurum chinense DC.; Scutellaria baicalensis Georgi; Bos taurus (bile calculus processed); Viola philippica Cav.; Patrinia scabiosaefolia Fisch. ex Trevir.; Houttuynia cordata Thunb.; Phragmites australis (Cav.) Trin. ex Steud.; Vigna angularis (Willd.) Ohwi & H.Ohashi | Apiaceae; Lamiaceae; Bovidae-derived; Violaceae; Caprifoliaceae; Saururaceae; Poaceae; Fabaceae | Root; root; gallstone derivative; whole herb; whole herb; whole herb; rhizome; seed | CP-listed proprietary formula (limited disclosure; not fully standardized in trials) | Yabao Pharmaceutical / Beijing University TCM affiliated pharma factories | NR (industrial proprietary formulation) | Not reported | NR | POWO (for botanical taxa); MPNS not cited in trials | Partial |
| Marketed Chinese patent medicines | Lanqin Oral Liquid / 蓝芩口服液 | Proprietary multi-herbal TCM formula (patent medicine) | 6 | Oral liquid | Oral | 10 mL per dose, BID–TID (mostly TID) | 5–7 days | Isatis root; Scutellaria root; Gardenia fruit; Phellodendron bark; (sometimes added auxiliary herbs not consistently reported) | Isatis indigotica Fort.; Scutellaria baicalensis Georgi; Gardenia jasminoides Ellis; Phellodendron chinense Schneid. | Brassicaceae; Lamiaceae; Rubiaceae; Rutaceae | Root; root; fruit; bark | CP-listed proprietary formula (limited disclosure; not fully standardized in trials) | Yangtze River Pharmaceutical Group | NR (industrial proprietary formulation) | Not reported | NR | Not reported (no POWO/MPNS cited) | Partial |
| Marketed Chinese patent medicines | Ganjie Bingmei Tablet / 甘桔冰梅片 | Proprietary multi-herbal TCM formula (patent medicine) | 5 | Tablet | Oral | 0.4 g per dose (2 tablets), QID (most trials); sometimes 2 tablets QID / 5–7 days equivalent | 5–14 days | Platycodon root; Scutellaria root; Prunus armeniaca kernel? (not consistently reported); Gardenia fruit; Phellodendron bark; Mentha; Cicada slough; Mume fruit; Glycyrrhiza root; (some formulations include Isatis/other auxiliaries not consistent across trials) | Platycodon grandiflorus (Jacq.) A.DC.; Scutellaria baicalensis Georgi; Gardenia jasminoides J.Ellis; Phellodendron chinense C.K.Schneid.; Mentha haplocalyx Briq.; Cryptotympana atrata (Fabricius); Prunus mume (Siebold) Siebold & Zucc.; Glycyrrhiza uralensis Fisch. | Platycodonaceae; Lamiaceae; Rubiaceae; Rutaceae; Lamiaceae; Cicadidae (insect); Rosaceae; Fabaceae | Root; root; fruit; bark; aerial parts; insect; fruit; root | CP-listed Chinese patent medicine (approved NMPA; proprietary formulation) | Chongqing Huasen Pharmaceutical Co.,Ltd. | NR (industrial proprietary formulation) | Not reported | NR | POWO (for botanical taxa); MPNS not cited in trials | Partial |
| Marketed Chinese patent medicines | Qingyan Dripping Pills / 清咽滴丸 | Proprietary multi-herbal TCM formula (patent medicine) | 4 | dripping pills | Oral | Most: 6pills TID (×3/day);alternative: 4–5pills 4–6 times/day;pediatric/adapted regimens not specified | 5–7 days | Buffalo horn/bovis calculus (artificial cow bezoar); Mentha; Indigo naturalis; Borneol; Terminalia chebula; Glycyrrhiza uralensis; (plus auxiliary excipients depending on manufacturer) | Moschus/Bovis calculus; Mentha haplocalyx Briq.; Strobilanthes cusia (Nees) Kuntze; Borneolum syntheticum; Terminalia chebula Retz.; Glycyrrhiza uralensis Fisch. | Rutaceae/Lamiaceae/Acanthaceae (varies by herb) | Fruit/leaf/mineral/extract mixtures | CP-listed Chinese patent medicine (NMPA approved) | Tianjin Sixth Chinese Medicine Factory / Tianjin Zhongxin Pharmaceutical Group | NR (industrial proprietary formulation) | Not reported | NR | POWO (for botanical taxa); MPNS not cited in trials | Partial |
| Marketed Chinese patent medicines | Houyanqing Granules / 喉咽清颗粒 | Proprietary multi-herbal TCM formula (patent medicine) | 4 | Rod-shaped granules | Oral | Most studies: 6 g per dose, 3 times/day (tid); pediatric study: weight/age adjusted not specified | 5–7 days | Turmeric-homologous clearing heat formula: Radix Achyranthis, Herba Spilanthes/Erigeron-like species, Plantaginis Semen/Herba, Lophatheri Herba mixed regional nomenclature in CN literature | Achyranthes bidentata Blume (or related Achyranthes spp.); unclear “Malan grass” taxonomic resolution (likely Kalimeris/Erigeron complex in practice); Plantago asiatica L.; Centipeda minima / Inula-related taxa reported variably | Amaranthaceae; Asteraceae; Plantaginaceae; Asteraceae | Food/medicinal aerial parts & roots (varies by herb) | CP-listed Chinese patent medicine (NMPA approved) | Hunan Shidai Yangguang Pharmaceutical / standardized GMP manufacturers | NR (industrial proprietary formulation) | Not reported | NR | POWO (for botanical taxa); MPNS not cited in trials | Partial |
| Marketed Chinese patent medicines | Tanreqing Injection / 痰热清注射液 | Proprietary multi-herbal TCM formula (patent medicine) | 4 | Injection solution | IV infusion | IV: 10–20 ml + 250–500 ml glucose or saline once daily | 3–5 days | Huangqin, Xiongdanfen (bear bile powder), Shanyangjiao, Jinyinhua, Lianqiao | Scutellaria baicalensis Georgi; Ursus species (bile); Capra hircus Linnaeus (horn); Lonicera japonica Thunb.; Forsythia suspensa (Thunb.) Vahl | Lamiaceae; Ursidae (animal-derived); Bovidae; Caprifoliaceae; Oleaceae | Root; bile; horn; flower; fruit | CP-listed Chinese patent medicine (NMPA approved) | Shanghai Kaibao Pharmaceutical Co., Ltd. (Shanghai Kaibao Drug Industry) | NR (industrial proprietary formulation) | Not reported | NR | POWO (for botanical taxa); MPNS not cited in trials | Partial |
| Marketed Chinese patent medicines | Lianhua Qingwen Capsule / 连花清瘟胶囊 | Proprietary multi-herbal TCM formula (patent medicine) | 3 | Capsule | Oral | 2–4 capsules per dose, TID (most studies: 2–3 capsules TID) | 3–5 days | Fructus Forsythiae; Flos Lonicerae; Herba Ephedrae (processed); Semen Armeniacae Amarum; Gypsum Fibrosum; Radix Isatidis; Herba Houttuyniae; Herba Pogostemonis; Radix et Rhizoma Rhei; Rhodiola rosea; Menthol; Glycyrrhiza uralensis | Forsythia suspensa (Thunb.) Vahl; Lonicera japonica Thunb.; Ephedra sinica Stapf; Prunus armeniaca L.; Gypsum (mineral); Isatis indigotica Fort.; Houttuynia cordata Thunb.; Pogostemon cablin (Blanco) Benth.; Rheum palmatum L.; Rhodiola rosea L.; Glycyrrhiza uralensis Fisch. | Oleaceae;Caprifoliaceae;Ephedraceae;Rosaceae;mineral;Brassicaceae;Saururaceae;Lamiaceae;Polygonaceae;Crassulaceae;Fabaceae | Flower; flower; stem; seed; mineral; root; whole herb; aerial part; root; root; root | CP-listed Chinese patent medicine (NMPA approved) | Shijiazhuang Yiling Pharmaceutical Co., Ltd. | NR (industrial proprietary formulation) | Not reported | NR | POWO (for botanical taxa); MPNS not cited in trials | Partial |
| Marketed Chinese patent medicines | Shuanghuanglian Injection/双黄连注射液 | Proprietary multi-herbal TCM formula (patent medicine) | 3 | Injection | IV infusion | IV: 60 mg/kg or 1–2 ampules daily | 3–7 days | Flos Lonicerae; Fructus Forsythiae; Radix Scutellariae (core 3-herb formula) | Lonicera japonica Thunb.; Forsythia suspensa (Thunb.) Vahl; Scutellaria baicalensis Georgi | Caprifoliaceae; Oleaceae; Lamiaceae | Flower; fruit; root | CP-listed Chinese patent medicine (NMPA approved multi-formulation system) | Harbin Pharmaceutical Group / multiple provincial manufacturers (varies by study) | NR (industrial proprietary formulation) | Not reported | NR | POWO (for botanical taxa); MPNS not cited in trials | Partial |
| Marketed Chinese patent medicines | Xiyanping Injection / 喜炎平注射液 | Proprietary multi-herbal TCM formula (patent medicine) | 3 | Injection | IV infusion | IV: 250 mg in 250 mL NS once daily | 3–5 days | Andrographis paniculata extract (andrographolide derivatives / sulfonated andrographolides) | Andrographis paniculata (Burm.f.) Nees | Acanthaceae | Whole herb (aerial parts) | CP-listed Chinese patent injection (NMPA approved sterile injection) | Hubei Huarun Sanjiu / multiple GMP pharmaceutical manufacturers | NR (industrial proprietary formulation) | Not reported | NR | POWO (for botanical taxa); MPNS not cited in trials | Partial |
| Marketed Chinese patent medicines | Zhongtong'an Capsule / 肿痛安胶囊 | Proprietary multi-herbal TCM formula (patent medicine) | 3 | Capsule | Oral | 0.56 g per dose, TID (3 times/day); some combined therapy with antibiotics (e.g., amoxicillin 0.5 g QID) | 5–7 days | Typhonii Rhizoma (Baifuzi), Arisaematis Rhizoma (Tiannanxing), Bombyx Batryticatus (Jiangcan), Gastrodiae Rhizoma (Tianma), Saposhnikoviae Radix (Fangfeng), Notopterygii Rhizoma (Qianghuo), Angelicae Dahuricae Radix (Baizhi), Panax notoginseng (Sanqi) | Typhonium giganteum Engl.; Arisaema erubescens (Wall.) Schott; Bombyx batryticatus; Gastrodia elata Blume; Saposhnikovia divaricata (Turcz.) Schischk.; Notopterygium incisum Ting ex H.T. Chang; Angelica dahurica (Hoffm.) Benth. & Hook.f. ex Franch. & Sav.; Panax notoginseng (Burkill) F.H.Chen | Araceae; Araceae; Bombycidae (animal); Orchidaceae; Apiaceae; Apiaceae; Apiaceae; Araliaceae | Root/rhizome/insect body/root/root/root/root/root | CP-listed Chinese patent medicine (NMPA approved proprietary formulation) | Hebei Aoxing Group Pharmaceutical Co., Ltd. | NR (industrial proprietary formulation) | Not reported | NR | POWO (for botanical taxa); MPNS not cited in trials | Partial |
| individualized prescriptions | Qingrun Decoction / 清润汤 | Multi-herb individualized TCM formula | 3 | Nebulized decoction solution | Inhalation via ultrasonic nebulizer | 20 mL nebulized solution; 30 min per session; 2 times/day | 5 days per course | Lonicerae Japonicae Flos, Platycodonis Radix, Scutellariae Radix, Sophorae Tonkinensis Radix, Belamcandae Rhizoma, Radix Puerariae, Panax quinquefolius, Glycyrrhizae Radix et Rhizoma | Lonicera japonica Thunb.; Platycodon grandiflorus (Jacq.) A.DC.; Scutellaria baicalensis Georgi; Sophora flavescens Aiton; Belamcanda chinensis (L.) DC.; Pueraria lobata (Willd.) Ohwi; Panax quinquefolius L.; Glycyrrhiza uralensis Fisch. | Caprifoliaceae; Campanulaceae; Lamiaceae; Fabaceae; Iridaceae; Fabaceae; Araliaceae; Fabaceae | Rhizome/root/flower/root/rhizome/root/root/root | None (custom hospital decoction; not pharmacopeial standardized product) | Hospital pharmacy / outpatient TCM decoction unit | Not reported | Not reported | NR | POWO (for botanical taxa); MPNS not cited in trials | Partial |
| Individualized prescriptions | Gancao Jiegeng Shegan Decoction / 甘草桔梗射干汤加味 | Multi-herb individualized TCM decoction (modified classical formula) | 2 | Granule decoction / water decoction for oral administration | Oral | 200 mL decoction daily; 100 mL per dose; 2 times/day (morning + evening) | 7 days | Glycyrrhizae Radix et Rhizoma; Platycodonis Radix; Belamcandae Rhizoma; Scutellariae Radix; Pinelliae Rhizoma; Scrophulariae Radix; Taraxaci Herba | Glycyrrhiza uralensis Fisch.; Platycodon grandiflorus (Jacq.) A.DC.; Belamcanda chinensis (L.) DC.; Scutellaria baicalensis Georgi; Pinellia ternata (Thunb.) Breit.; Scrophularia ningpoensis Hemsl.; Taraxacum mongolicum Hand.-Mazz. | Fabaceae; Campanulaceae; Iridaceae; Lamiaceae; Araceae; Scrophulariaceae; Asteraceae | Root/rhizome/root/root/rhizome/root/herb | None (hospital-based individualized prescription; no pharmacopeial product) | Hospital TCM pharmacy | Not reported | Not reported | NR | POWO (for botanical taxa); MPNS not cited in trials | Partial |
| Individualized prescriptions | Shufeng Qingre Decoction / 疏风清热汤 | Multi-herb individualized TCM decoction | 2 | Decoction (water decoction) | Oral | 300 mL/day; 100 mL per dose | 7 days | Glycyrrhizae Radix; Saposhnikoviae Radix; Lonicerae Flos; Forsythiae Fructus; Platycodonis Radix; Belamcandae Rhizoma; Scutellariae Radix; Scrophulariae Radix; Taraxaci Herba; Arctii Fructus; Phragmitis Rhizoma; Paeoniae Radix Rubra | Glycyrrhiza uralensis Fisch.; Saposhnikovia divaricata (Turcz.) Schischk.; Lonicera japonica Thunb.; Forsythia suspensa (Thunb.) Vahl; Platycodon grandiflorus (Jacq.) A.DC.; Belamcanda chinensis (L.) DC.; Scutellaria baicalensis Georgi; Scrophularia ningpoensis Hemsl.; Taraxacum mongolicum Hand.-Mazz.; Arctium lappa L.; Phragmites australis (Cav.) Trin. ex Steud.; Paeonia lactiflora Pall. | Asteraceae; Apiaceae; Caprifoliaceae; Oleaceae; Campanulaceae; Iridaceae; Lamiaceae; Scrophulariaceae; Asteraceae; Asteraceae; Poaceae; Paeoniaceae | Root/rhizome/flower/fruit/root/rhizome/root/root/herb/fruit/rhizome/root | None (hospital decoction; non-commercial individualized prescription) | Hospital TCM pharmacy | Not reported | Not reported | NR | POWO (for botanical taxa); MPNS not cited in trials | Partial |
| Individualized prescriptions | Qingyan Chubi Decoction / 清咽除痹汤 | Multi-herb individualized TCM decoction | 2 | Decoction | Oral | 100 mL twice daily | 5–10 days | Pulverized/recorded formula: Taraxaci Herba; Rehmanniae Radix; Paeoniae Radix Rubra; Moutan Cortex; Bupleuri Radix; Scutellariae Radix; Armeniacae Semen Amarum (fried); Scrophulariae Radix; Forsythiae Fructus; Isatidis Radix; Citri Reticulatae Pericarpium; Fritillariae Thunbergii Bulbus; Adenophorae Radix / Glehniae Radix ; Glycyrrhizae Radix | modifications: Glycyrrhiza uralensis Fisch.; Rehmannia glutinosa Libosch.; Paeonia lactiflora Pall.; Paeoniae suffruticosa Andrews; Bupleurum chinense DC.; Scutellaria baicalensis Georgi; Prunus armeniaca L.; Scrophularia ningpoensis Hemsl.; Forsythia suspensa (Thunb.) Vahl; Isatis indigotica Fort.; Citrus reticulata Blanco; Fritillaria thunbergii Miq.; Adenophora stricta Miq. / Glehnia littoralis F.Schmidt ex Miq. | Asteraceae; Orobanchaceae; Paeoniaceae; Paeoniaceae; Apiaceae; Lamiaceae; Rosaceae; Scrophulariaceae; Oleaceae; Brassicaceae; Rutaceae; Liliaceae; Campanulaceae/Apiaceae; Fabaceae | Root/herb/fruit/bulb/rhizome | None (hospital TCM individualized prescription) | Hospital preparation | Not reported | Not reported | NR | POWO (for botanical taxa); MPNS not cited in trials | Partial |
| Individualized prescriptions | Qingyan Li’ge Formula Granules / 清咽利膈配方颗粒 | Multi-herb TCM granule formula | 2 | Granules | Oral | 1 packet per dose, 100 mL warm water, 3 times/day (tid) | 5 days | Core composition (from both trials): Flos Lonicerae; Fructus Forsythiae; Herba Schizonepetae; Radix Saposhnikoviae; Folium Menthae; Fructus Arctii; Radix Scrophulariae; Fructus Gardeniae; Radix Scutellariae; Rhizoma Coptidis; Radix Isatidis; Radix Platycodonis; Natrii Sulfas (Xuanming powder); Radix Glycyrrhizae | + Botanical names: Lonicera japonica Thunb.; Forsythia suspensa (Thunb.) Vahl; Schizonepeta tenuifolia Briq.; Saposhnikovia divaricata (Turcz.) Schischk.; Mentha canadensis L.; Arctium lappa L.; Scrophularia ningpoensis Hemsl.; Gardenia jasminoides J.Ellis; Scutellaria baicalensis Georgi; Coptis chinensis Franch.; Isatis indigotica Fort.; Platycodon grandiflorus (Jacq.) A.DC.; Glycyrrhiza uralensis Fisch. | Asteraceae / Lamiaceae / Brassicaceae / Scrophulariaceae / Rubiaceae / Fabaceae / Campanulaceae etc. | Flower/fruit/root/rhizome/herb | None (TCM hospital granule preparation; no pharmacopeia compliance stated) | Hospital prepared proprietary granules (TCM hospital formulation; no manufacturer consistency reported) | Not reported | Not reported | NR | POWO (for botanical taxa); MPNS not cited in trials | Partial |
| hospital preparations | Yanhouqing Buccal Tablets / 咽喉清口含片 | Multi-center RCT (Phase II + Phase III clinical trials) | 2 | Buccal tablets | Oral | 0.4 g per dose (2 tablets) | 5 days | Lonicera japonica Thunb.; Platycodon grandiflorus (Jacq.) A.DC.; Belamcanda chinensis (L.) DC.; Fritillaria spp.; Scrophularia ningpoensis Hemsl.; Terminalia chebula Retz.; Siraitia grosvenorii (Swingle); Scutellaria baicalensis Georgi (partial inferred in formula class); etc. | Lonicera japonica Thunb.; Platycodon grandiflorus (Jacq.) A.DC.; Belamcanda chinensis (L.) DC.; Fritillaria spp.; Scrophularia ningpoensis Hemsl.; Terminalia chebula Retz.; Siraitia grosvenorii (Swingle); Scutellaria baicalensis Georgi (partial inferred in formula class); etc. | Lonicerae / Campanulaceae / Iridaceae / Liliaceae / Scrophulariaceae / Combretaceae / Cucurbitaceae etc. | Flower/root/rhizome/fruit/resin etc. | none (hospital preparation, no pharmacopeial monograph stated) | Guangzhou Kangyuan Pharmaceutical Co., Ltd. | Not reported | Not reported | NR | POWO (for botanical taxa); MPNS not cited in trials | Partial |
